# Peptides from Mackerel Skin Prepared by the Mixed Proteases: Fractionation, Characterization and Bioactivities

**DOI:** 10.3390/foods14061009

**Published:** 2025-03-16

**Authors:** Yichen Zhu, Leyi Zheng, Lei Gu, Yijiao Qiao, Changhua Xu

**Affiliations:** 1 College of Food Science and Technology, Shanghai Ocean University, Shanghai 201306, China; m220301006@st.shou.edu.cn (Y.Z.); m230300967@st.shou.edu.cn (L.Z.); 2 Shanghai Engineering Research Center of Aquatic-Product Processing and Preservation, Shanghai 201306, China; m230300927@st.shou.edu.cn (L.G.); m240401158@st.shou.edu.cn (Y.Q.); 3 Laboratory of Quality and Safety Risk Assessment for Aquatic Products on Storage and Preservation (Shanghai), Ministry of Agriculture, Shanghai 201306, China; 4 National R&D Branch Center for Freshwater Aquatic Products Processing Technology (Shanghai), Shanghai 201306, China

**Keywords:** mackerel, peptides, targeted enzymatic extraction, high-value utilization, biological activities

## Abstract

Mackerel is widely favored by consumers as a high-yield, delicious marine fish. However, by-products generated during its processing, such as fish skins, are often underutilized, resulting in significant resource waste. This study aimed to extract high-activity mackerel protein peptides (HA-MPPs) from mackerel skins through targeted enzymatic hydrolysis (using a composite protease). The peptides were purified using ultrafiltration and HPLC, and their biological activity was evaluated through infrared imaging and antioxidant assays. Mass spectrometry identified the main peptide fragments (P1, P2, and P3). The optimal conditions for enzymatic hydrolysis were 0.22% enzyme concentration, a 2.03 h hydrolysis time, 55.05 °C, and a 1:3 solid-to-liquid ratio, yielding 59.66%. Infrared imaging showed that HA-MPPs exhibited significant biological repair activities, penetrating the hair cuticle to restore keratin and enhance hair strength. Additionally, antioxidant assays confirmed their abilities to reduce oxidative damage. This study presents a novel method for the targeted enzymatic extraction of HA-MPPs from mackerel by-products and the high-value utilization of their biological activity. It also demonstrates the potential of these peptides in hair repair, providing a theoretical foundation for the future development of hair care products with reparative functions.

## 1. Introduction

With the fast-tracked progress in aquatic product processing and comprehensive utilization in China, the fisheries industry has become one of the three pillar industries [1]. The high-value utilization of by-products from aquatic product processing has gradually become a key area of focus [2]. By-products such as fish bones and skins are often regarded as waste in the fishery processing industry, leading to resource wastage and environmental pollution due to their ineffective utilization [3]. As a result, the development of environmentally friendly and sustainable methods for the processing and high-value utilization of these by-products has become an urgent issue to address [4]. In recent years, the aquaculture industry of mackerel (*Scomber japonicus*) has experienced rapid growth, particularly after the policy lifting in 2016, which resulted in a surge in market demand. However, during the processing of mackerel, large amounts of by-products, such as fish skins, are discarded. These by-products are rich in collagen, making them ideal raw materials for the extraction of collagen peptides [5]. Collagen peptides, as hydrolysis products, have low molecular weights, high absorption rates, and significant physiological functions and nutritional value. Specifically, collagen peptides derived from aquatic sources have gained attention as an ideal alternative to traditional animal-derived collagen due to their low allergenicity and high safety [5,6].

Currently, the preparation methods of fish-derived protein peptides mainly include chemical hydrolysis, microwave-assisted extraction, chemical synthesis, and enzymatic hydrolysis. Chemical hydrolysis is simple and cost-effective but may lead to modifications in amino acids and changes in functionality. Additionally, the process is relatively difficult to control, which limits its effectiveness in obtaining peptides with high biological activity [7]. While microwave-assisted extraction improves extraction efficiency, the localized high temperatures may cause peptide degradation, affecting their biological activity [8,9,10]. Although chemical synthesis allows for the precise production of target peptides, it is inefficient, costly, and not suitable for large-scale applications [11]. Therefore, enzymatic hydrolysis, with its mild reaction conditions and higher peptide yields, has gradually become the preferred method for preparing fish-derived protein peptides [12]. By optimizing enzymatic hydrolysis conditions, such as enzyme concentration, reaction time, and temperature, the yield and activity of peptides can be significantly enhanced [13].

Furthermore, peptide purification is a key step in improving the quality and activity of fish-derived protein peptides. Traditional purification methods, such as membrane filtration [14], gel filtration chromatography [15,16], and ion-exchange chromatography [17,18], are commonly used in the preliminary separation process. However, these methods often fail to provide sufficient purity, and some may involve contamination issues, limiting their effectiveness in large-scale applications [19]. As a result, reverse-phase high-performance liquid chromatography (HPLC), with its high resolution and excellent operability, has been widely employed for the separation and purification of bioactive peptides [20]. Additionally, liquid chromatography–tandem mass spectrometry (LC-MS/MS) technology, as an efficient tool for peptide sequence identification and bioactivity assessment, further enhances the depth and precision of peptide research [21].

Based on the background outlined above, this study focused on the extraction of fish skin peptides from mackerel skin using enzymatic hydrolysis, with peptide yield serving as the optimization indicator. Building on this, the influence of various factors, including enzyme concentration, hydrolysis duration, and the solid-to-liquid ratio, was evaluated. The extraction and enzymatic hydrolysis parameters for mackerel skin peptides (HA-MPPs) were then optimized through Box–Behnken design and response surface methodology (RSM). Following this, HA-MPPs were purified via reverse-phase high-performance liquid chromatography (HPLC), and their bioactivity and peptide sequences were determined using multi-molecular infrared imaging, antioxidant assays, and liquid chromatography–tandem mass spectrometry (LC-MS/MS). This study explored the high-value utilization of mackerel by-products and provides experimental data to support their potential bioactivity and broad applications.

## 2. Materials and Methods

### 2.1. Materials

The mackerel skin, a by-product of the *Scomber japonicus* processing, was provided by Zhongyang Ecological Fishery Co., Ltd., Nantong, China, caught from the East China Sea. The skins were frozen and stored at −20 °C until they were required for the study. The compound protease, composed of *alkaline protease* and *trypsin*, with an enzyme activity of 1.5 AU/g, was obtained through fermentation of *Bacillus amyloliquefaciens* and *Bacillus licheniformis*, and was supplied by Novozymes Biotechnology Co., Ltd., Beijing, China Concentrated hydrochloric acid was obtained from Kerlings Reagents Co., Ltd., Shanghai, China, and phenol was acquired from McLin Bio-Tech Co., Ltd., Shanghai, China. Reagents such as dithiothreitol, iodoacetamide, and ammonium bicarbonate were purchased from Sigma-Aldrich, St. Louis, MI, USA. Acetonitrile and methanol were sourced from Thermo Fisher Scientific, Waltham, MA, USA. The C18 analytical column (4.6 mm × 250 mm, 5 μm) came from Agilent Technologies, Santa Clara, CA, USA, and the C18 preparative column (19 mm × 150 mm, 5 μm) was provided by Waters Corporation, Milford, MA, USA. The freezing embedding medium (OCT) was purchased from SAKURA, Tokyo, Japan. The ZnSe transmission slides (13 mm × 2 mm), as a component of the ATR imaging system’s transmission attachment, were used for infrared imaging analysis.

### 2.2. Single-Factor Experimental Design

#### 2.2.1. Enzyme Concentration

Mackerel skin was selected as the raw material, and water was added at a liquid-to-solid ratio of 3:1. Compound protease (a mixture of alkaline protease and trypsin) was applied at various concentrations (0, 0.05%, 0.1%, 0.15%, 0.2%, 0.25%, 0.3%, and 0.35%, relative to the quantity of raw material). Enzymatic hydrolysis, with an enzyme activity ratio of 1:1, was conducted at 50 °C for 4 h, followed by enzyme deactivation at 90 °C for 15 min to halt the reaction. Samples were then collected, and the peptide yield was quantified using the trichloroacetic acid (TCA) precipitation method [22].

#### 2.2.2. Liquid-to-Solid Ratio

Mackerel skin was used as the raw material, and a compound enzyme solution (0.2% enzyme concentration with an enzyme activity ratio of 1:1) was applied. A range of liquid-to-solid ratios (1:1, 2:1, 3:1, 4:1, 5:1, and 6:1) was tested. The enzymatic hydrolysis was performed at 50 °C for 4 h, followed by enzyme deactivation at 90 °C for 15 min. Samples were taken for peptide yield analysis.

#### 2.2.3. Hydrolysis Duration

Mackerel skin was employed as the raw material, and 0.2% compound enzyme solution (enzyme activity ratio of 1:1) was added, maintaining a 3:1 ratio of liquid to solid. Enzymatic hydrolysis was conducted at 50 °C for various durations: 0, 0.5, 1, 1.5, 2, 2.5, and 3 h, followed by enzyme deactivation at 90 °C for 15 min to terminate the reaction. The peptide yield was determined by sampling at different time intervals.

#### 2.2.4. Hydrolysis Temperature

Mackerel skin was selected as the starting material, and a 0.2% compound enzyme solution was introduced, maintaining a liquid-to-solid ratio of 3:1. Enzymatic hydrolysis was conducted at different temperatures (35 °C, 40 °C, 45 °C, 50 °C, 55 °C, 60 °C, and 65 °C) for a fixed period of 4 h, followed by enzyme deactivation at 90 °C for 15 min. The peptide yield was assessed from the resulting hydrolysates.

#### 2.2.5. pH of Hydrolysis Reaction

Mackerel skin was utilized as the raw material, with the addition of 0.2% compound enzyme solution (enzyme activity ratio of 1:1) and a liquid-to-solid ratio of 3:1. Enzymatic hydrolysis was performed at varying pH levels (4, 5, 6, 7, 8, and 9) at 50 °C for 4 h, with subsequent enzyme deactivation at 90 °C for 15 min. The peptide yield was quantified by collecting and analyzing samples at different pH conditions.

### 2.3. Response Surface Methodology Optimization

The peptide yield from mackerel skin was used as the response variable. Building on the results of the single-factor experiments, the impacts of enzyme concentration, temperature, and hydrolysis time were assessed. The Box–Behnken design, executed with Design Expert 8.0.6.1 software, was employed to fine-tune the extraction parameters [23,24]. The factors and levels for the optimization experiments are shown in Table 1.

### 2.4. Optimization of Mackerel Skin Peptide Extraction Process

The extraction process of mackerel skin peptides was optimized utilizing the data from preliminary single-factor and response surface trials. Distilled water was added to the fish skin at a fish skin-to-water ratio of 1:3 (*w*/*v*) and subjected to a constant temperature extraction at 90 °C for 3 h. Subsequently, 2% compound protease was added, and enzymatic hydrolysis was implemented at 55 °C and pH 7.0, with 120 rpm water bath shaking for 2 h. After enzymatic hydrolysis, the enzyme was deactivated at 90 °C for 15 min. After the hydrolysate cooled to room temperature for 20 min, it was centrifuged at 8000 rpm for 15 min at 4 °C. The resulting supernatant was transferred into a 500 mL Erlenmeyer flask, and 1.5% activated carbon was added. The mixture was subjected to decolorization in a 60 °C water bath for 30 min, followed by filtration to obtain mackerel skin protein peptides (HA-MPPs). The peptides were then separated into three molecular weight fractions (10,000–3000 Da, 3000–1000 Da, and <1000 Da) using ultrafiltration [25]. The fraction with a molecular weight below 1000 Da was chosen as the target peptide, freeze-dried, and stored at −4 °C for subsequent use.

### 2.5. Amino Acid Analysis of HA-MPPs

The amino acid determination method for HA-MPPs followed the national standard GB5009.124-2016 [26]. A total of 15 mg of HA-MPP lyophilized powder was weighed into a hydrolysis tube, and 10 mL of 6 mol/L hydrochloric acid was added. After cooling at 4 °C for 5 min, three drops of phenol were added to the mixture for color development. The mixture was then vacuum-sealed and hydrolyzed at 110 °C for 22 h. The solution was then cooled and diluted to 50 mL; 1 mL of the filtrate was absorbed into an evaporating dish and vacuum-dried at 50 °C. The residue was dissolved in 1 mL of distilled water and vacuum-dried again until completely evaporated. The residue was then dissolved in 2 mL of pH 2.2 sodium citrate solution and filtered through a 0.22 μm aqueous-phase membrane. The filtrate was collected in an autosampler vial and stored at −20 °C for amino acid analysis using an amino acid analyzer (model LA-8080).

### 2.6. Purification of HA-MPPs by HPLC

HA-MPPs were prepared at a concentration of 20 mg/mL and passed through a 0.22 μm filter. The resulting solution was purified using a Waters e2695 HPLC system equipped with a UV detector, autosampler, and other modules. The separation was carried out using a SunFire C18 OBD preparative column (19 × 150 mm, 5 μm), with an injection volume of 1 mL. The mobile phase consisted of solvent A (0.05% trifluoroacetic acid, TFA, in water) and solvent B (acetonitrile). A gradient elution was performed at a flow rate of 4 mL/min for 35 min. The elution program was as follows: from 0 to 25 min, solvent A was decreased linearly from 95% to 70%, while solvent B was increased from 5% to 30%; from 25 to 28 min, solvent A was decreased linearly from 70% to 55%, while solvent B was increased from 30% to 45%; from 28 to 35 min, solvent A was increased from 45% to 95%, while solvent B was decreased from 55% to 5%. The eluate was monitored at 220 nm, and fractions were collected based on the chromatographic profile [27]. These fractions were subsequently freeze-dried for bioactivity assessment.

### 2.7. Bioactivity Evaluation of HA-MPPs Fractions

The bioactivity of the collected fractions was assessed using a Spotlight 400 Fourier Transform Infrared (FTIR) Imaging Spectrometer, Thermo Fisher Scientific, Waltham, MA, USA. This technique enabled the identification of the fraction with the most significant repair effect, which was selected for further bioactivity evaluation.

### 2.8. Purity Determination of HA-MPPs

The purity of the bioactive peptide fractions was analyzed using an analytical HPLC system [28]. The fractions were prepared at a concentration of 10 mg/mL and passed through a 0.22 μm filter. Purification was carried out using a C18 analytical column (250 × 4.6 mm), and 20 μL of each sample was injected. The mobile phase consisted of solvent A (0.05% TFA in water) and solvent B (acetonitrile). A gradient elution was conducted at a flow rate of 0.833 mL/min for 40 min with the following conditions: 0–5 min, 80% solvent A; 5–30 min, 80–55% solvent A; 30–35 min, 55–70% solvent A; 35–40 min, 70–95% solvent A. The eluate was monitored at 220 nm, and fractions were collected based on the chromatographic data. The fractions collected were then freeze-dried for subsequent analysis.

### 2.9. LC-MS/MS Analysis of the Amino Acid Sequence of High-Activity HA-MPPs

LC-MS/MS was employed to further separate and identify the component with the highest bioactivity. Dithiothreitol (DTT) was utilized to reduce disulfide bonds within the peptides, facilitating complete denaturation and enabling the separation of peptide chains. Following reduction, iodoacetamide (IAM) was introduced to alkylate the cysteine residues, thereby preventing the reformation of disulfide bonds and stabilizing the peptide structure. The sample was subsequently buffered with ammonium bicarbonate (NH_4_HCO_3_) to maintain an optimal pH environment for enzymatic digestion, ensuring compatibility with LC-MS/MS analysis. After LC analysis, the component was introduced into a Q-Exactive Plus mass spectrometer for sequencing. The EASY-nLC 1200 system coupled with a C18 micro-column (75 μm × 15 cm, 3 μm) was used for the analysis [29]. The chromatographic conditions were set as follows: mobile phase A was a 0.1% formic acid aqueous solution, while mobile phase B consisted of an 80% acetonitrile aqueous solution containing 0.1% formic acid. The elution gradient was configured as follows: 0–3 min, 2–6% B; 3–42 min, 6–20% B; 42–47 min, 22–35% B; 47–48 min, 35–100% B; 48–60 min, 100% B. Amino acid sequence analysis was carried out using the PEAKS Studio 10.0 search engine.

### 2.10. Synthesis of High-Activity Mackerel Protein Peptides

The peptides identified through mass spectrometry were produced by Gil Biochemical Co., Ltd., Shanghai, China. The purity and molecular weight of the produced peptides were assessed using RP-HPLC with an AU-2000 LC system (4.6 mm × 250 mm, 5 μm) and analyzed by mass spectrometry (Agilent 61258B, Santa Clara, CA, USA.).

### 2.11. Permeability Assessment of HA-MPPs

The biological activity of HA-MPPs was characterized using infrared ATR imaging. The damaged hair samples treated with P1, P2, and P3 in Section 2.11 were analyzed via ATR imaging. The samples were embedded in OCT, processed into frozen sections, and subjected to ATR imaging to obtain protein optical imaging and infrared absorption maps of the hair cross-sections. These results were compared with those of normal hair samples. The parameters for spectral acquisition were configured as follows: a wavenumber range from 4000 to 750 cm^−1^, a resolution of 4 cm^−1^, a pixel size of 6.25 μm, and 16 scans for each pixel.

### 2.12. HA-MPP Antioxidant Activity Assay

#### 2.12.1. Total Antioxidant Activity Test

The overall antioxidant activity was measured using the ABTS approach with an ABTS assay kit [30]. According to the kit instructions, a working solution was prepared. The antioxidant capacity of a Trolox standard solution was first measured to generate a standard curve. Then, the working solution was incubated with different types of HA-MPPs (crude peptides, P1, P2, P3) at room temperature for 6 min, and the absorbance was measured at 414 nm [31].

#### 2.12.2. Total Superoxide Dismutase (SOD) Activity Assay

The total SOD activity was measured using an SOD Assay Kit, Yuan Ye Biotechnology Co., Ltd., Shanghai, China. Following the kit instructions, the working solution was prepared and incubated with different types of HA-MPPs (crude peptides, P1, P2, P3) at 37 °C for 30 min. The absorbance was then measured at 560 nm [32].

#### 2.12.3. Hydroxyl Radical (OH-) Scavenging Activity Assay

The scavenging ability of hydroxyl radicals was assessed using a hydroxyl radical scavenging activity assay kit. A working solution was prepared according to the kit instructions, and the solution was pre-warmed at 37 °C for 3 min. Different types of HA-MPPs (crude peptides, P1, P2, P3) were then mixed with the working solution and incubated at 37 °C for 20 min. The absorbance was measured at 550 nm at room temperature.

### 2.13. Data Processing

GraphPad Prism 9.5.1 and SPSS 27.0 software were used for the creation of relevant data charts and statistical analysis, including variance analysis.

## 3. Results and Discussion

### 3.1. Analysis of the Results from the Single-Factor Experiment

**Figure 1:**
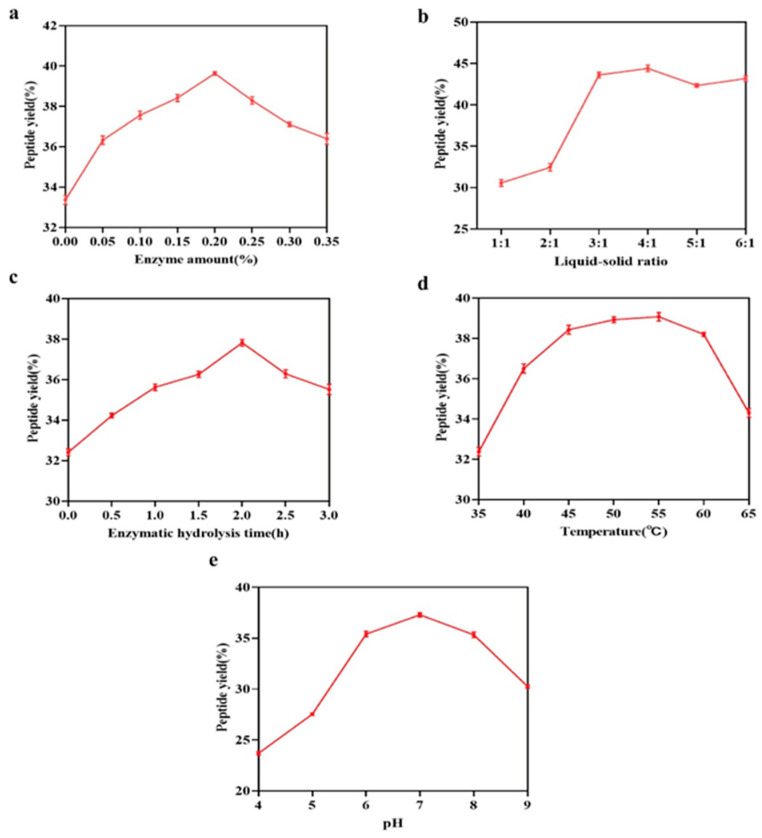
Single-factor experimental analysis of the extraction process of HA-MPPs: (**a**) effect of enzyme amount; (**b**) effect of liquid–solid ratio; (**c**) effect of enzymatic hydrolysis time; (**d**) effect of enzymatic hydrolysis temperature; (**e**) effect of pH.

The single-factor experimental analysis of the HA-MPP extraction process is shown in Figure 1. Figure 1a illustrates the effect of enzyme concentration on peptide yield, indicating that as the enzyme concentration increased, the yield rose, reaching a maximum of 39.63% (mean value) at 0.2% composite protease, after which the yield began to decline. To achieve a high peptide yield while minimizing costs, an enzyme concentration of approximately 0.2% was recommended for further studies. Figure 1b demonstrates the impact of the liquid-to-solid ratio, showing that when the ratio reached 3:1, the peptide yield stabilized and remained consistent, providing the most effective extraction. Figure 1c reveals the influence of hydrolysis time, with 2 h being the optimal duration, as extending the reaction time did not significantly increase the yield. In Figure 1d, the impact of temperature on the enzymatic reaction is demonstrated, with the reaction rate rising with increasing temperature until it reached a maximum. However, further increases in temperature caused protease denaturation, reducing the effective enzyme concentration and slowing the reaction rate, suggesting an optimal hydrolysis temperature around 55 °C. Finally, Figure 1e presents the effect of pH, where the highest peptide yield (37.30%) occurred at pH 7, indicating that a neutral pH was the optimal condition for the enzymatic reaction.

### 3.2. Response Surface Optimization of HA-MPP Preparation Process Conditions

**Figure 2:**
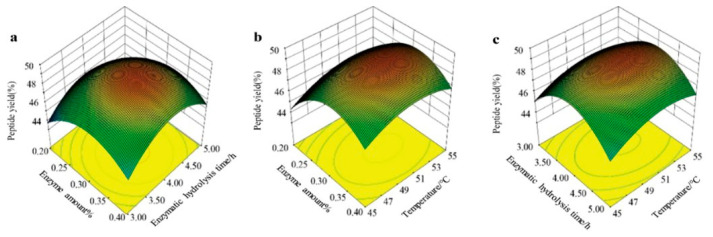
Response surface 3D plot of the effect of enzymatic hydrolysis conditions on peptide yield (%). (**a**) Enzyme amount and Enzymatic hydrolysis time; (**b**) Enzyme amount and Temperature; (**c**) Enzymatic hydrolysis time and Temperature.

Taking the HA-MPP yield as the response variable, and according to the results from the single-factor tests and a review of relevant literature on marine fish-derived peptide extraction, enzyme amount, enzymatic hydrolysis time, and temperature were identified as the three main factors. A Box–Behnken design was employed to optimize the extraction conditions using response surface methodology, with Design Expert 8.0.6.1 software. The experimental setup and optimization outcomes are presented in Figure 2 and Table 2, and the analysis of variance along with the significance results are shown in Table 3.

A regression analysis was performed on the data, and a regression equation was obtained as follows: Peptide yield = 49.10 + 0.81X_1_ + 0.094X_2_ + 0.44X_3_ + 0.20X_1_X_2_ − 0.017X_1_X_3_ + 0.085X_2_X_3_ − 2.26X_1_^2^ − 2.38X_2_^2^ − 1.10X_3_^2^. This equation was employed to represent the correlation between the factors and the response value. The results indicate that the linear relationship was significant (R² = 0.8679), with the model reaching a significant level (*p* < 0.05). Furthermore, the lack-of-fit F-value was not significant, suggesting that the model fit well and can be used. The three factors affecting peptide yield—enzyme amount, enzymatic hydrolysis temperature, and enzymatic hydrolysis time—had significance levels smaller than 0.01, indicating that their effects were highly significant. In summary, through single-factor experiments and response surface analysis, the optimal enzymatic hydrolysis conditions for mackerel skin peptides were determined as follows: an enzyme amount of 0.22%, an enzymatic hydrolysis time of 2.03 h, an enzymatic hydrolysis temperature of 55.05 °C, and a solid-to-liquid ratio (*w*/*v*) of 1:3. In these ideal conditions, the predicted peptide yield was 59.98%, while the experimental yield was 59.66% (mean value), with no significant difference between the experimental result and the response surface model prediction, indicating a good agreement.

### 3.3. Establishment of Targeted Preparation Method for HA-MPPs

**Figure 3:**
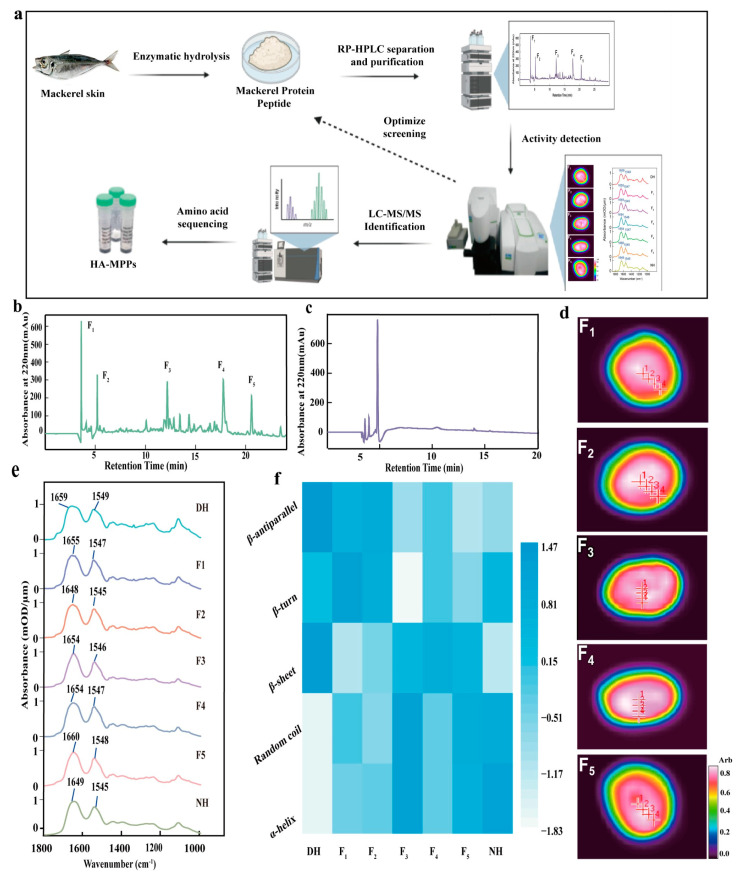
Preparation and characterizations of HA-MPPs; (**a**) preparation process of HA-MPPs (**b**) purification chromatogram by RP-HPLC, (**c**) analytical RP-HPLC profile of F3, (**d**) infrared imaging of protein in transverse section of five damaged hair (DH) samples treated with protein peptides (F1–F5) separated by RP-HPLC, (**e**) average spectra from infrared imaging of protein in different points of normal hair (NH), damaged hair (DH), and DH treated with HA-MPPs (F1–F5) (three parallel replicates), (**f**) heat map of standardized values for the protein secondary structure of NH, DH, and DH treated with HA-MPPs (F1–F5).

Drawing from the outcomes above, this study designed a series of targeted preparation methods for HA-MPPs (Figure 3a) and collected five different components (F1–F5) (Figure 3b). The biological activity of each component was evaluated by its ability to repair damaged hair (DH), and infrared transmission imaging was employed to comprehensively reveal their chemical information and spatial distribution [33,34]. The infrared images of the proteins in the hair fibers clearly displayed their internal structure, with point 1 representing the hair medulla, points 2 and 3 representing the cortex, and point 4 representing the cuticle (Figure 3d). From the infrared images, it was evident that proteins were primarily concentrated in the central part of the hair. Notably, after treatment with F1-F5, the characteristic peaks of the DH samples exhibited a red shift, approaching the characteristic peaks of normal hair (NH), indicating that HA-MPPs had good repair activity for DH (Figure 3e).

The various conformations of protein peptide chains directly determined their physiological functions and activities [35,36]. In this research, the amide I region of the protein’s secondary structure was examined to compare the activity of the F1–F5 components. To evaluate the biological activity of these components, we assessed their ability to repair damaged hair fibers. This approach was chosen because the main goal of this research was to determine high-activity peptides that could effectively repair hair damage. After treatment with F1–F5, the α-conformation of the DH samples increased, with the F3 treatment group showing a 5.25% increase, approaching that of normal hair (Figure 3f). The F3 component, isolated by RP-HPLC (Figure 3b), contained fewer polar peptides, indicating that it had more hydrophobic units, which enhances its affinity for hair keratin (H-KRT). Therefore, the F3 component was selected for further purification and analysis.

### 3.4. Establishment of the Mass Spectrometry Sequencing Results of HA-MPPs

The amino acid sequences of the F3 fraction components were identified using nano LC-Q-TOF-MS, followed by further analysis using PEAKS Studio 10.0 software. Several amino acid sequences and related information were successfully obtained. Based on the relative peak area and abundance values, three representative peptide sequences were selected as the main peptide sequences of HA-MPPs (see Table 4): P1 (*m*/*z* 336), P2 (*m*/*z* 407), and P3 (*m*/*z* 393). The corresponding primary and secondary mass spectrometry results are provided in the Supplementary Materials.

### 3.5. ATR Imaging Analysis of the Biological Activity of HA-MPPs

Based on previous permeability experiments (detailed in the Supplementary Materials), we found that P1, P2, and P3 exhibited good permeability for damaged hair. To explore their bioactivity and the changes in the internal chemical composition, further analysis was needed to understand the specific chemical changes occurring inside the hair. Infrared imaging could provide in situ distribution of chemical components within the hair. By collecting infrared images from different sections of the hair, the changes occurring during peptide repair could be analyzed more effectively.

**Figure 4:**
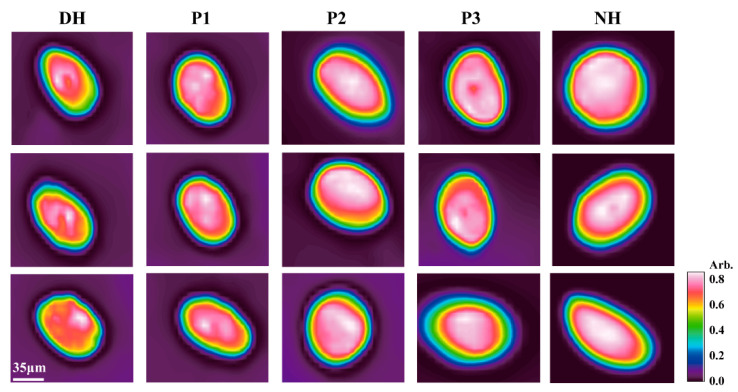
Chemical infrared imaging of protein in hair treated with different HA-MPPs.

**Figure 5:**
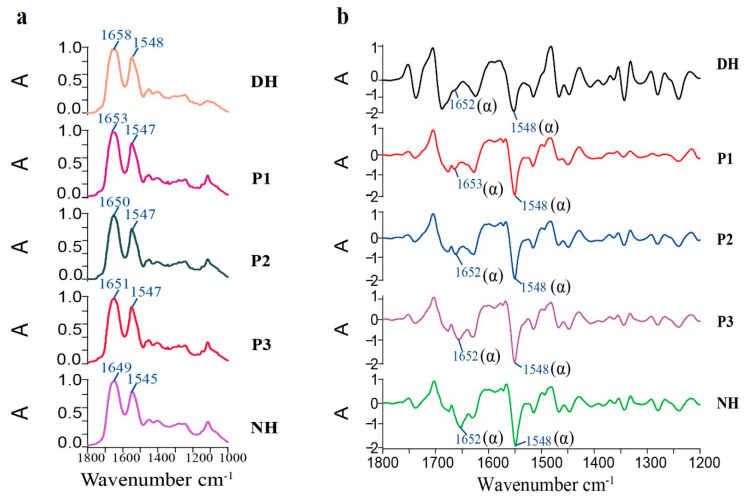
One-dimensional infrared spectrum (**a**) and second derivative spectrum (**b**) extracted from the protein region of infrared imaging (average spectrum after triplicate measurements).

As shown in Figure 4 and Table 5, the infrared distribution of proteins in healthy hair generally covered the entire cross-section. In unhealthy hair, the distribution area was reduced, the intensity was weaker, and it was concentrated in the medulla. After treatment with synthetic peptides, the infrared absorption intensity distribution increased by 18% to 20%. As illustrated in Figure 5a, after treatment with P1, P2, and P3, the high-wavenumber region of the one-dimensional protein spectrum gradually shifted toward the low-wavenumber region, exhibiting a red shift and approaching the characteristic protein bands of healthy hair. Figure 5b and Table 6 further indicate that, compared to DH, the secondary structure of the P1, P2, and P3 treatment groups underwent significant changes, particularly in the α-helix conformation, making it more similar to that of normal hair. These results further confirm that the internal protein structure of the hair changed after treatment, collectively supporting the hypothesis that HA-MPPs promoted protein penetration and structural reorganization within hair fibers. This suggests that HA-MPPs not only adsorbed onto the hair surface to repair and improve its morphology and structure but also penetrated the hair fibers, significantly restoring the keratin in unhealthy hair.

### 3.6. Antioxidant Evaluation of HA-MPPs

**Figure 6:**
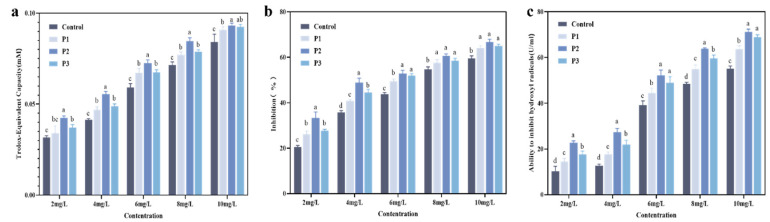
Evaluation of antioxidant properties of different HA-MPPs; (**a**) ABTS^+^ radical scavenging activity, (**b**) detection of SOD activity, (**c**) hydroxyl radical scavenging ability. (Different low case letters above columns indicate statistical differences at *p* < 0.05).

Reactive oxygen species (ROS), such as hydrogen peroxide, hydroxyl radicals, and superoxide anions, are key contributors to oxidative stress and inflammation in the body [37]. The antioxidant activity of HA-MPPs was evaluated, and the results are shown in Figure 6. The ability of different HA-MPPs components to scavenge ABTS+ radicals increased with the concentration of the sample, with the P2 component at 10 mg/mL exhibiting the strongest antioxidant capacity. As the concentration increased, the rate of radical scavenging followed the order: P2 > P3 > P1 > crude peptides. As indicated in Figure 6b, SOD activity showed a positive correlation with the HA-MPPs components overall. The antioxidant capacity differences were minimal between 6–8 mg/mL, while beyond 8 mg/mL, the activity began to plateau. Figure 6c demonstrates that different HA-MPP components exhibited strong scavenging ability against hydroxyl radicals, with scavenging rates positively correlated to their concentrations. The inhibitory effect on hydroxyl radicals was weak at 2 mg/mL and 4 mg/mL, but significantly improved after reaching 6 mg/mL, with a continuous increase in inhibition observed at higher concentrations.

In summary, various HA-MPP components (crude peptides, P1, P2, P3) exhibit substantial scavenging ability against both ABTS+ and hydroxyl radicals, along with enhanced antioxidant enzyme SOD activity, indicating their promising antioxidant potential. This suggests that after enzymatic hydrolysis, the low-molecular-weight hydrolysates or peptides may more effectively react with free radicals [38]. These hydrolysates likely generate ionized amino groups, carboxyl groups, and other hydrogen donors, as well as peptides rich in hydrophobic amino acids and active amino acid sequences, thereby enhancing antioxidant activity. HA-MPPs hold significant promise in delaying lipid oxidation and reducing protein degradation by quenching or scavenging ROS, with considerable potential for applications in bioactivity and medical repair fields.

## 4. Conclusions

Based on the experimental results, this study proposes an innovative method for extracting mackerel protein peptides from mackerel by-products (fish skin) using targeted enzymatic hydrolysis. Through single-factor experiments and response surface optimization, the optimal extraction parameters were determined: enzyme concentration of 0.22%, hydrolysis time of 2.03 h, hydrolysis temperature of 55.05 °C, and a solid-to-liquid ratio of 1:3, resulting in a peptide yield of 59.66%. Subsequently, multi-stage separation and purification techniques, including ultrafiltration and HPLC, were employed to successfully isolate HA-MPPs. Further mass spectrometry sequencing identified the main peptide fragments of HA-MPPs, such as P1, P2, and P3. Through infrared imaging, the purified HA-MPPs demonstrated significant bio-repair activity, effectively penetrating the hair cuticle to repair damaged keratin and enhance the strength and resilience of hair fibers. Further antioxidant assessments revealed that HA-MPPs exhibited strong antioxidant activity, helping to reduce oxidative hair damage and maintain hair health.

In conclusion, this study not only provided a new strategy for the high-value utilization of mackerel by-products but also demonstrated the potential of HA-MPPs in hair repair. By combining targeted enzyme extraction with bioactivity screening, a marine protein peptide with good biological activity was successfully developed. This research lays a theoretical foundation for the future development of hair care medical products and offers a new direction for the high-value utilization of fish by-products.

## Figures and Tables

**Table 1:** Factors and levels of the experiment.

Factors	Levels		
	−1	0	1
X_1_ enzyme amount/%	0.2	0.3	0.4
X_2_ enzymatic hydrolysis time/h	1	2	3
X_3_ temperature/°C	45	50	55

**Table 2:** Response surface optimization experimental design and results.

Run	X_1_: Enzyme Amount/%	X_2_: Enzymatic Hydrolysis Time/h	X_3_: Temperature/°C	HA-MPPs Yield/%
1	0.2	2	45	43.26%
2	0.3	2	50	49.50%
3	0.3	3	45	46.29%
4	0.3	2	50	48.99%
5	0.3	2	50	49.45%
6	0.4	1	50	45.02%
7	0.3	2	50	49.00%
8	0.2	1	50	43.97%
9	0.2	2	55	49.98%
10	0.4	3	50	45.37%
11	0.3	3	55	45.39%
12	0.3	2	50	48.14%
13	0.3	1	45	46.03%
14	0.4	2	45	45.38%
15	0.3	1	55	44.79%
16	0.4	2	55	47.88%
17	0.2	3	50	43.51%

**Table 3:** Analysis of variance (ANOVA) for the regression model.

Source	SS	DF	MS	F-Value	*p*-Value
model	62.80	9	6.98	5.11	0.0214 *
X_1_	5.22	1	5.22	3.82	0.0916
X_2_	0.070	1	0.070	0.051	0.8270
X_3_	1.58	1	1.58	1.15	0.3184
X_1_X_2_	0.16	1	0.16	0.12	0.7391
X_1_X_3_	0.12	1	0.12	0.087	0.7764
X_2_X_3_	0.029	1	0.029	0.021	0.8884
X_1_^2^	21.50	1	21.50	15.74	0.0054 **
X_2_^2^	23.79	1	23.79	17.42	0.0042 **
X_3_^2^	5.11	1	5.11	3.74	0.0942
residual	9.56	7	1.37		
Omission item	7.83	3	2.61	6.04	0.0574
pure error	1.73	4	0.43		
total dispersion	72.36	16			

Note: * Significant (*p* < 0.05); ** highly significant (*p* < 0.01).

**Table 4:** Summary of mass spectrometry sequencing results.

Sequences	−10 lgP	Length	Mass	*m*/*z*	Area Sample
P1	21.62	3	335.1845	336.1921	2.58 × 10^10^
P2	24.69	4	406.2216	407.2295	2.40 × 10^9^
P3	23.96	4	392.2059	393.2138	2.38 × 10^9^

**Table 5:** Percentage distribution of protein infrared absorption intensity.

Groups	DH	P1	P2	P3	NH
absorption intensity	38% ± 6%	56% ± 3%	58% ± 2%	57% ± 4%	60% ± 7%

**Table 6:** Secondary structure analysis of P1-P3 treatments extracted from infrared imaging.

Secondary Structure	DH	P1	P2	P3	NH
a-helix	10.98	15.10	15.21	16.71	16.57
Random coil	13.79	15.16	14.67	16.31	16.08
β-sheet	35.18	30.59	31.20	32.20	30.45
β-turn	28.80	28.74	28.33	25.97	27.98
β-antiparallel	11.25	10.41	10.58	8.80	8.94

## Data Availability

The original contributions presented in this study are included in the article/Supplementary Materials. Further inquiries can be directed to the corresponding author.

## Supplementary Materials

The following supporting information can be downloaded at: https://www.mdpi.com/article/10.3390/foods14061009/s1, Figure S1: Fluorescence microscopy images of DH cross-sections after treatment with different fluorescently labeled synthetic peptides for 1 h, 2 h, and 3 h (a) P1; (b) P2; (c) P3; Figure S2: Comparison of DH treated with HA MPPs (P1, P2, P3) and healthy hair (NH) (a) Gloss; (b) Friction coefficient; (c) Tensile strength.

## Abbreviations

The following abbreviations are used in this manuscript:

HA-MPPs	High-activity mackerel protein peptides
DH	Damaged hair
H-KRT	Hair keratin
ROS	Reactive oxygen species

## References

[1] Li J.R., Lu H.X., Zhu J.L., Wang Y.B., Li X.P. (2009). Aquatic products processing industry in China: Challenges and outlook. Trends Food Sci. Technol..

[2] Yuan Z., Ye X., Hou Z., Chen S. (2024). Sustainable utilization of proteins from fish processing by-products: Extraction, biological activities and applications. Trends Food Sci. Technol..

[3] Ozogul F., Cagalj M., Simat V., Ozogul Y., Tkaczewska J., Hassoun A., Kaddour A.A., Kuley E., Rathod N.B., Phadke G.G. (2021). Recent developments in valorisation of bioactive ingredients in discard/seafood processing by-products. Trends Food Sci. Technol..

[4] Olsen R.L., Toppe J., Karunasagar I. (2014). Challenges and realistic opportunities in the use of by-products from processing of fish and shellfish. Trends Food Sci. Technol..

[5] Pan J.F., Li Q., Jia H., Xia L.N., Jin W.G., Shang M.J., Xu C., Dong X.P. (2018). Physiochemical and functional properties of tiger puffer (*Takifugu rubripes*) skin gelatin as affected by extraction conditions. Int. J. Biol. Macromol..

[6] Ahmed M., Verma A.K., Patel R. (2020). Collagen extraction and recent biological activities of collagen peptides derived from sea-food waste: A review. Sustain. Chem. Pharm..

[7] Vijaykrishnaraj M., Prabhasankar P. (2015). Marine protein hydrolysates: Their present and future perspectives in food chemistry—A review. Rsc Adv..

[8] Grosso C., Valentao P., Ferreres F., Andrade P.B. (2015). Alternative and Efficient Extraction Methods for Marine-Derived Compounds. Mar. Drugs.

[9] Belanger J.M.R., Pare J.R.J. (2006). Applications of microwave-assisted processes (MAP™) to environmental analysis. Anal. Bioanal. Chem..

[10] Wang N., Zhu H., Wang M., Zhao S., Sun G., Li Z. (2024). Recent Advancements in Microwave-Assisted Extraction of Flavonoids: A Review. Food Bioprocess Technol..

[11] Zhou Y. (2014). The Potential Biomedical Application of Cyclopeptides from Marine Natural Products. Curr. Org. Chem..

[12] Cunha S.A., Pintado M.E. (2022). Bioactive peptides derived from marine sources: Biological and functional properties. Trends Food Sci. Technol..

[13] Wang X., Yu H., Xing R., Chen X., Liu S., Li P. (2017). Optimization of the Extraction and Stability of Antioxidative Peptides from Mackerel (*Pneumatophorus japonicus*) Protein. Biomed Res. Int..

[14] Hung T.S., Bilad M.R., Shamsuddin N., Suhaimi H., Ismail N.M., Jaafar J., Ismail A.F. (2022). Confounding Effect of Wetting, Compaction, and Fouling in an Ultra-Low-Pressure Membrane Filtration: A Review. Polymers.

[15] O’Fagain C., Cummins P.M., O’Connor B.F., Walls D., Loughran S.T. (2011). Gel-Filtration Chromatography. Protein Chromatography: Methods and Protocols.

[16] Bouvier E.S.P., Koza S.M. (2014). Advances in size-exclusion separations of proteins and polymers by UHPLC. Trac-Trends Anal. Chem..

[17] Zhao H.X., Gao R.C., Bai P. (2014). Advances in Boron Isotope Separation by Ion Exchange Chromatography. Asian J. Chem..

[18] Velayudhan A., Horvath C. (1988). Preparative chromatography of proteins—Analysis of the multivalent ion-exchange formalism. J. Chromatogr..

[19] Wang X., Yu H., Xing R., Li P. (2017). Characterization, Preparation, and Purification of Marine Bioactive Peptides. BioMed Res. Int..

[20] Singh B.P., Vij S., Hati S. (2014). Functional significance of bioactive peptides derived from soybean. Peptides.

[21] Shaik M.I., Sarbon N.M. (2022). A Review on Purification and Characterization of Anti-proliferative Peptides Derived from Fish Protein Hydrolysate. Food Rev. Int..

[22] Liu T. (2006). Effect of phosphorylation on the enzymatic hydrolysis of Iow-value fish protein. Sci. Technol. Food Ind..

[23] Sonawane S.K., Arya S.S. (2017). Bioactive *L acidissima* protein hydrolysates using Box-Behnken design. 3 Biotech.

[24] Wang Z.Y., Zhao Y.Y., Su T.T. (2015). Extraction and antioxidant activity of polysaccharides from *Rana chensinensis* skin. Carbohydr. Polym..

[25] Wolfram L.J. (2003). Human hair: A unique physicochemical composite. J. Am. Acad. Dermatol..

[26] (2016). National Food Safety Standard: Determination of Amino Acids in Foods.

[27] Ennaas N., Hammami R., Beaulieu L., Fliss I. (2015). Purification and characterization of four antibacterial peptides from protamex hydrolysate of Atlantic mackerel (*Scomber scombrus*) by-products. Biochem. Biophys. Res. Commun..

[28] Careri A., Mangia A. (2003). Analysis of food proteins and peptides by chromatography and mass spectrometry. J. Chromatogr. A.

[29] Hye Lim J., Liceaga A.M., Kyung Young Y. (2016). Purification, characterisation and stability of an antioxidant peptide derived from sandfish (Arctoscopus japonicus) protein hydrolysates. J. Funct. Foods.

[30] Liu W., Gu R., Lin F., Lu J., Yi W., Cai M. (2012). Study on separation and purification of marine collagen oligopeptides and their antioxidant properties. Sci. Technol. Food Ind..

[31] Huang D.J., Ou B.X., Prior R.L. (2005). The chemistry behind antioxidant capacity assays. J. Agric. Food Chem..

[32] Sheng Y., Qiu Y.T., Wang Y.M., Chi C.F., Wang B. (2022). Novel Antioxidant Collagen Peptides of Siberian Sturgeon (*Acipenser baerii*) Cartilages: The Preparation, Characterization, and Cytoprotection of H_2_O_2_-Damaged Human Umbilical Vein Endothelial Cells (HUVECs). Mar. Drugs.

[33] Jiang C., Liu L., Li X.D., Ma L.Y., Du L.L., Zhao Y.B., Li D.H., Zhao W.L. (2018). Separation and purification of hypocholesterolaemic peptides from whey protein and their stability under simulated gastrointestinal digestion. Int. J. Dairy Technol..

[34] Zhang X.P., Li Y.R., Tao Y., Wang Y., Xu C.H., Lu Y. (2021). A novel method based on infrared spectroscopic inception-resnet networks for the detection of the major fish allergen parvalbumin. Food Chem..

[35] Feng Y.H., De Franceschi G., Kahraman A., Soste M., Melnik A., Boersema P.J., de Laureto P.P., Nikolaev Y., Oliveira A.P., Picotti P. (2014). Global analysis of protein structural changes in complex proteomes. Nat. Biotechnol..

[36] Qin Z., Buehler M.J. (2010). Molecular Dynamics Simulation of the α-Helix to β-Sheet Transition in Coiled Protein Filaments: Evidence for a Critical Filament Length Scale. Phys. Rev. Lett..

[37] Zou Z.Z., Chang H.C., Li H.L., Wang S.M. (2017). Induction of reactive oxygen species: An emerging approach for cancer therapy. Apoptosis.

[38] Zhang F., Qu J., Thakur K., Zhang J.G., Mocan A., Wei Z.J. (2019). Purification and identification of an antioxidative peptide from peony (*Paeonia suffruticosa* Andr.) seed dreg. Food Chem..

