# Supplementary material for: Peptides from Mackerel Skin Prepared by the Mixed Proteases: Fractionation, Characterization and Bioactivities
Source: Foods. 2025 Mar 16;14(6):1009. doi: 10.3390/foods14061009 (PMC11941859; doi:10.3390/foods14061009)
Supplement: Supplementary file 1 [file foods-14-01009-s001.zip › Mass spectrometry sequencing/P1, P2, P3 mass spectrometry spectra.pdf]

LGF (P1)

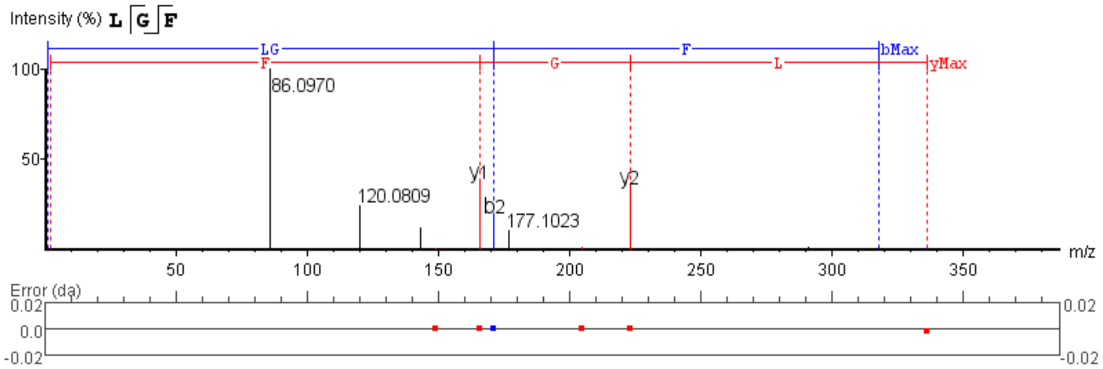

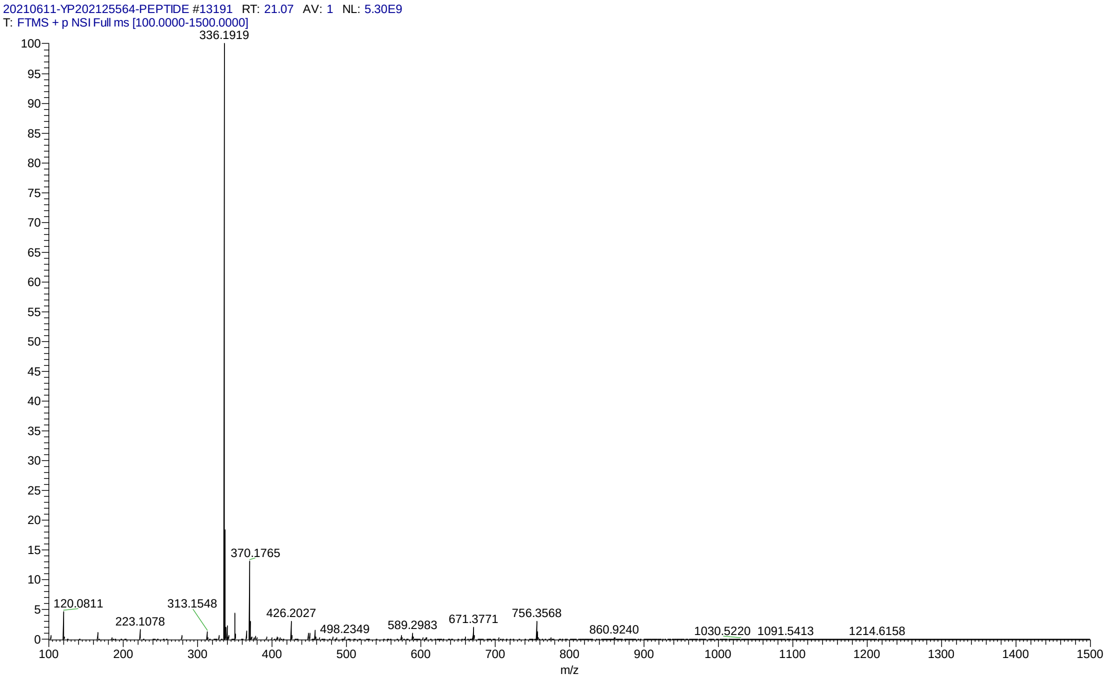

LAGF (P2)

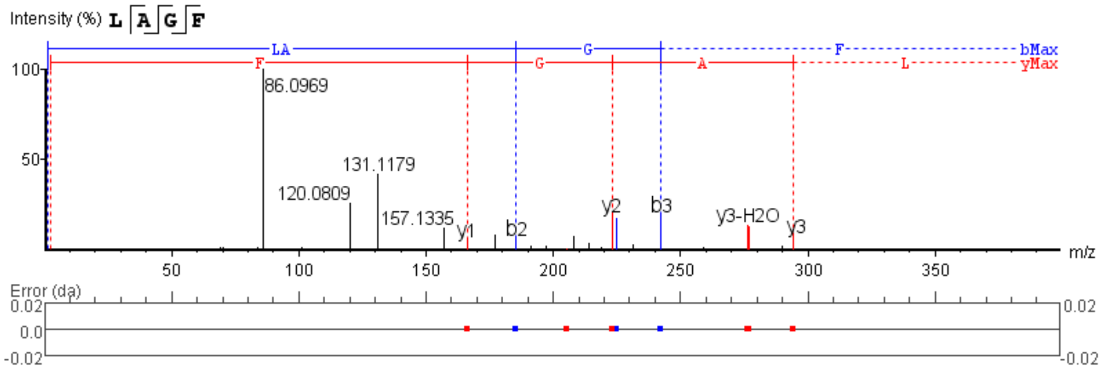

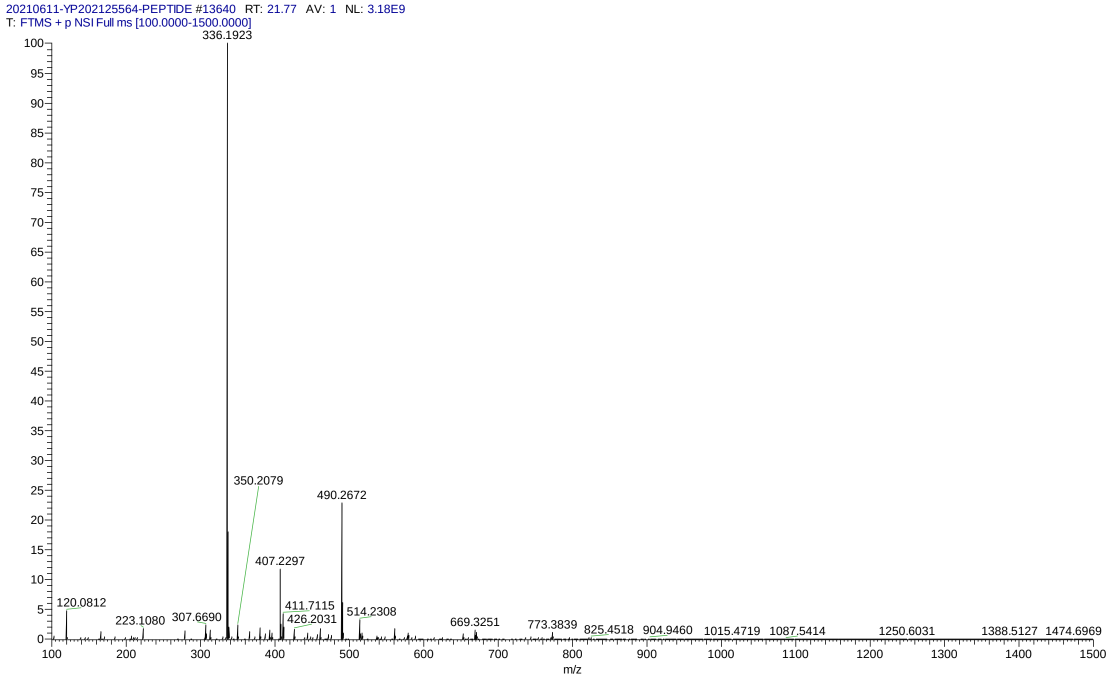
20210611-YP202125564-PEPTIDE #13640 RT: 21.77 AV: 1 NL: 3.18E9
T: FTMS + p NSI Full ms [100.0000-1500.0000]
336.1923
350.2079
490.2672
407.2297
120.0812
411.7115
426.2031
514.2308
223.1080
307.6690
669.3251
773.3839
825.4518
904.9460
1015.4719
1087.5414
1250.6031
1388.5127
1474.6969
m/z

LGGF (P3)

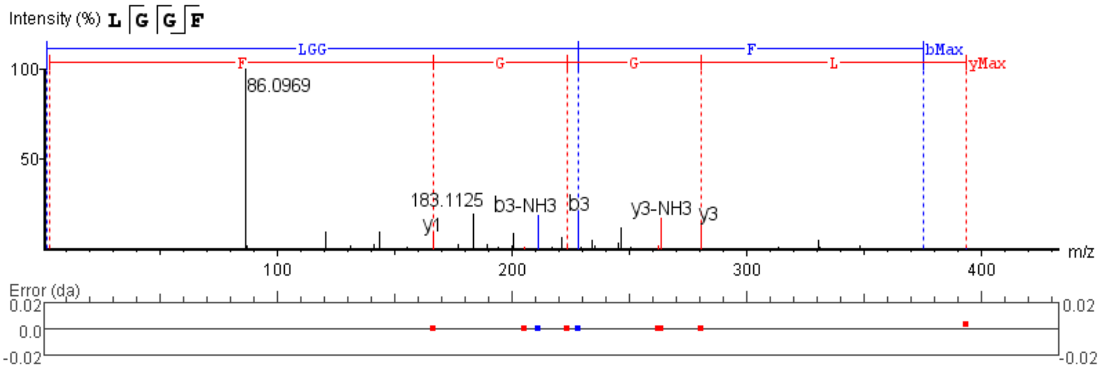
Intensity (%) L G G F
LGG
F
bMax
yMax
G
L
86.0969
183.1125
y1
b3-NH3
b3
y3-NH3
y3
m/z
Error (da)

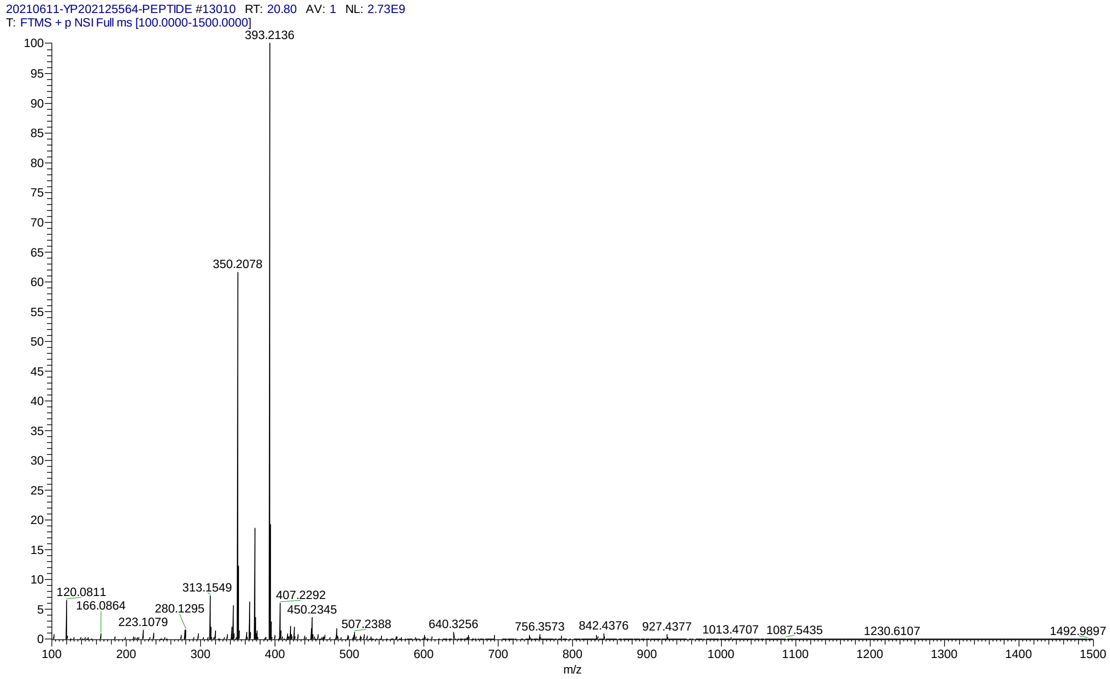
20210611-YP202125564-PEPTIDE #13010 RT: 20.80 AV: 1 NL: 2.73E9
T: FTMS + p NSI Full ms [100.0000-1500.0000]
393.2136
350.2078
120.0811
166.0864
223.1079
280.1295
313.1549
407.2292
450.2345
507.2388
640.3256
756.3573
842.4376
927.4377
1013.4707
1087.5435
1230.6107
1492.9897
m/z
