# Supplementary material for: Peptides from Mackerel Skin Prepared by the Mixed Proteases: Fractionation, Characterization and Bioactivities
Source: Foods. 2025 Mar 16;14(6):1009. doi: 10.3390/foods14061009 (PMC11941859; doi:10.3390/foods14061009)
Supplement: Supplementary file 1 [file foods-14-01009-s001.zip › Penetration assessment-Figure S1/Method, Results and Conclusion.pdf]

Materials: The fluorescently labeled peptides FITC-P1, FITC-P2, and FITC-P3 were synthesized by JIR Biochemical (Shanghai) Co., Ltd. The cryoembedding medium OCT was purchased from SAKURA, Japan. The cryostat (Model: CM1950) was obtained from LEICA, Germany. The fluorescence microscope (Model: OLYMPUS IX17) was purchased from Suzhou Jingtong Instrument Co., Ltd.

Methods: To investigate the permeability of P1, P2, and P3 in hair samples, these three peptides were fluorescently labeled with fluorescein isothiocyanate (FITC) and chemically synthesized. First, hair samples were washed with ultrapure water to remove surface impurities and naturally air-dried. The samples were then divided into groups and immersed in FITC-P1, FITC-P2, and FITC-P3 solutions for 1, 2, and 3 hours, respectively. After treatment, the hair fibers were washed three times, air-dried at room temperature for 12 hours, and stored in numbered containers. Next, the samples were embedded in OCT compound, placed at room temperature for 2 hours, and then frozen at -80°C for 3 hours. Using a cryostat, the embedded samples were sectioned into 10 μm thick slices and mounted on glass slides. Finally, fluorescence microscopy was used to observe the hair cross-sections, collect fluorescence images, and analyze the results.

Results and conclusions: The fluorescence microscopy images of hair fibers treated for 1 hour, 2 hours, and 3 hours (as shown in Figure S1) demonstrated effective penetration and affinity of HA-MPPs within the hair structure. When low molecular weight peptides were applied, a significant increase in peptide adsorption was observed, indicating strong penetration ability. Compared to P1, P2 and P3 exhibited a broader distribution within the damaged hair cortex, which can be attributed to differences in their amino acid sequences, leading to varying affinities for hair keratin. The effect of treatment duration on HA-MPPs' permeability was minimal, as peptide penetration was evident even after just 1 hour of treatment. As the treatment time increased, the amount of HA-MPPs adhering to the cuticle gradually increased, suggesting that these peptides began binding upon initial contact with damaged hair, penetrating the cortex and repairing the damaged keratin. Once internal repair was completed, the peptides predominantly adhered to the cuticle, further aiding in the repair of the protein structure in the hair. In conclusion, HA-MPPs effectively penetrated and adhered to the cuticle, thereby repairing the damaged hair structure.

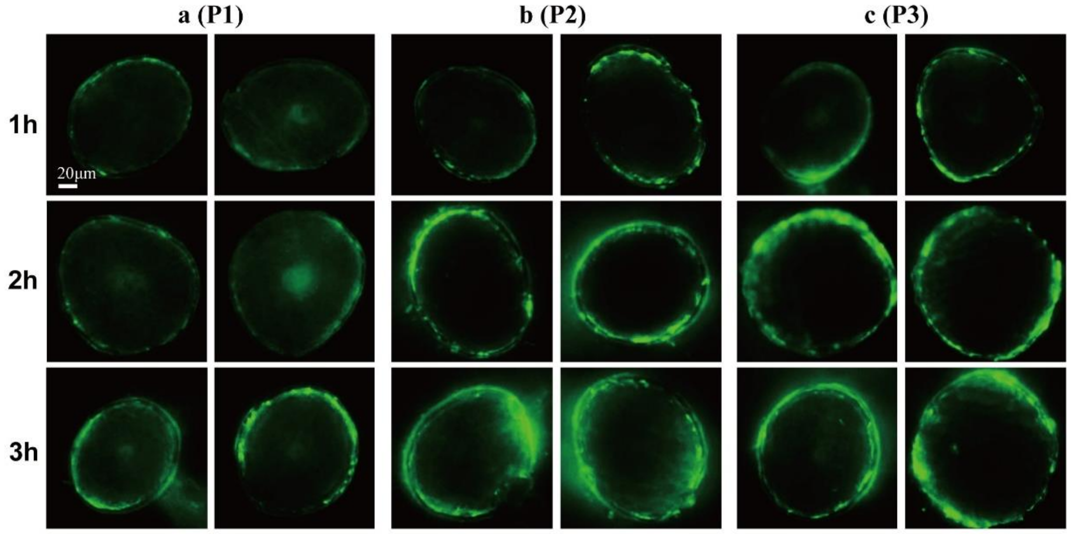

Figure S1 Fluorescence microscopy images of DH cross-sections after treatment with different fluorescently labeled synthetic peptides for 1h, 2h, and 3h (a) P1; (b) P2; (c) P3
