# Supplementary figures and images for: Peptides from Mackerel Skin Prepared by the Mixed Proteases: Fractionation, Characterization and Bioactivities
Source: Foods. 2025 Mar 16;14(6):1009. doi: 10.3390/foods14061009 (PMC11941859; doi:10.3390/foods14061009)

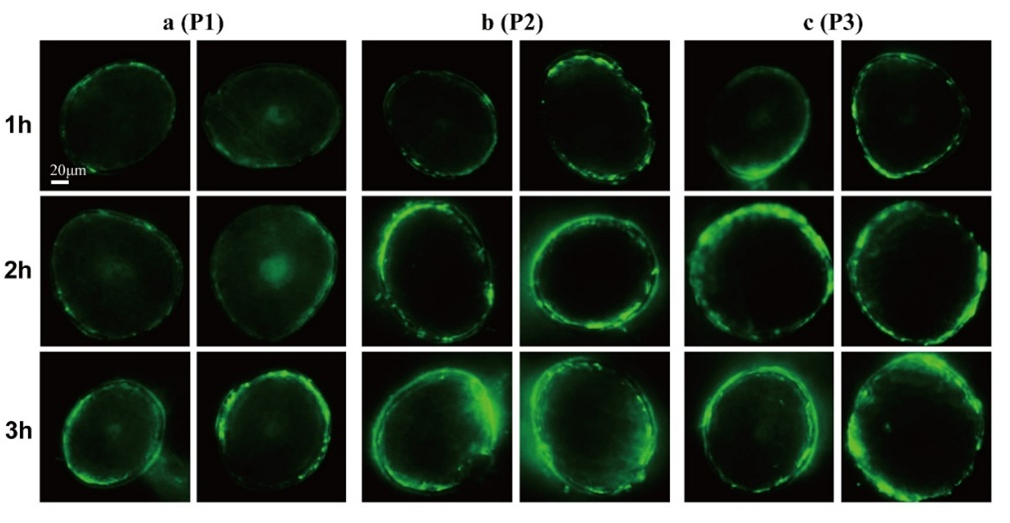

Supplement: Supplementary file 1 [file foods-14-01009-s001.zip › Figure S1.tif]

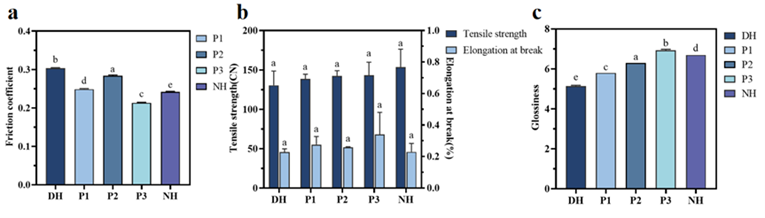

Supplement: Supplementary file 1 [file foods-14-01009-s001.zip › Figure S2.tif]

RT: 0.00 - 60.00

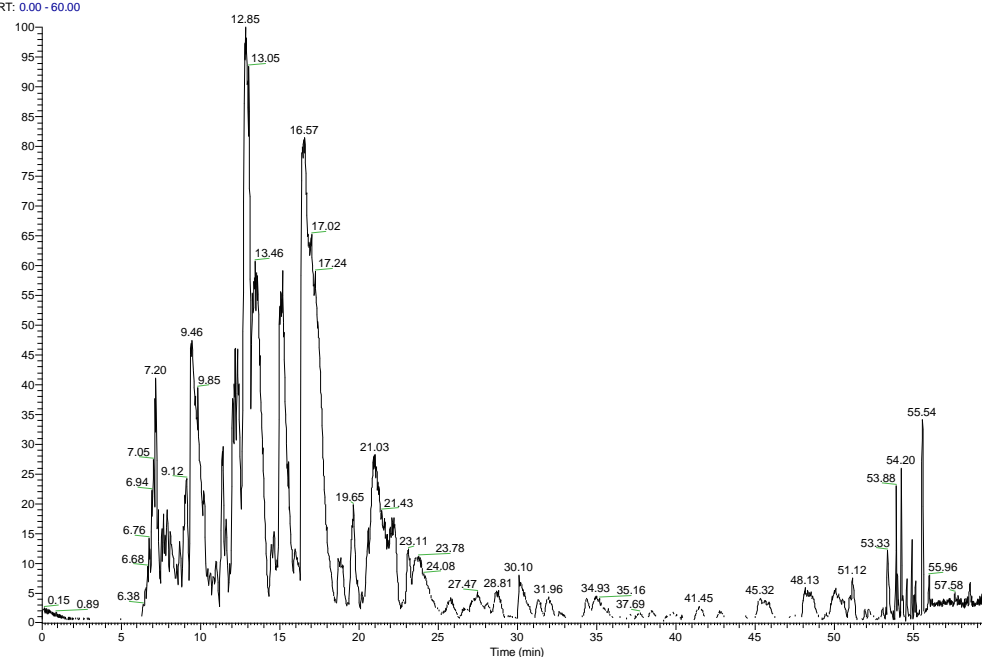

NL: 7.12E9  
Base Peak F: FTMS  
+ p NSI Full ms  
[100.0000-  
1500.0000] MS  
20210204-  
YP202104507-1

RT: 0.00 - 60.00

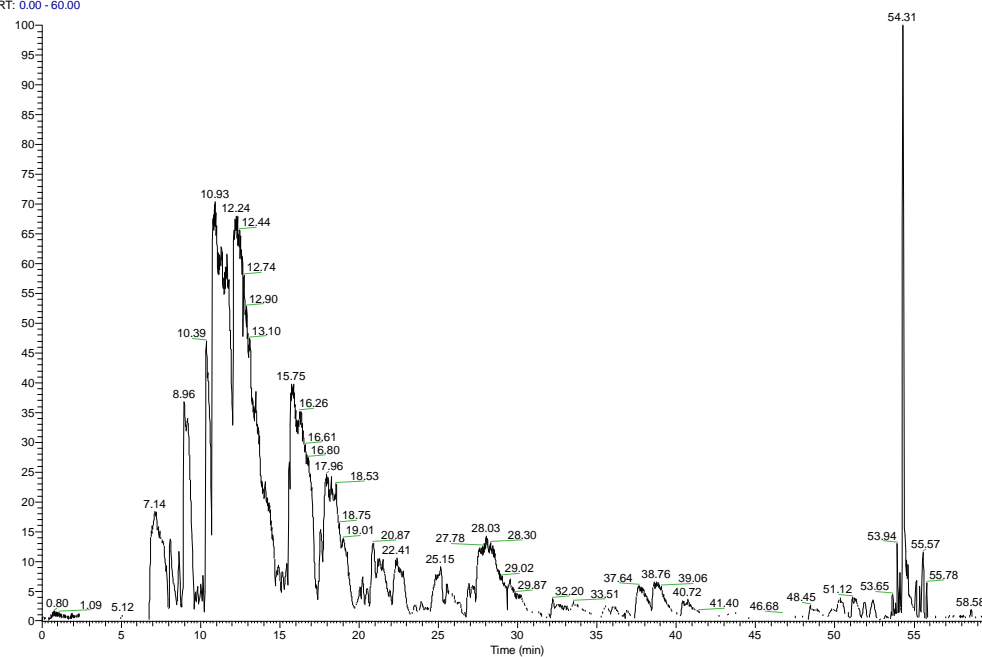

NL: 2.80E10  
Base Peak F: FTMS  
+ p NSI Full ms  
[100.0000-  
1500.0000] MS  
20210204-  
YP202104507-2

Supplement: Supplementary file 1 [file foods-14-01009-s001.zip › Mass spectrometry sequencing/Chromatogram.pdf]

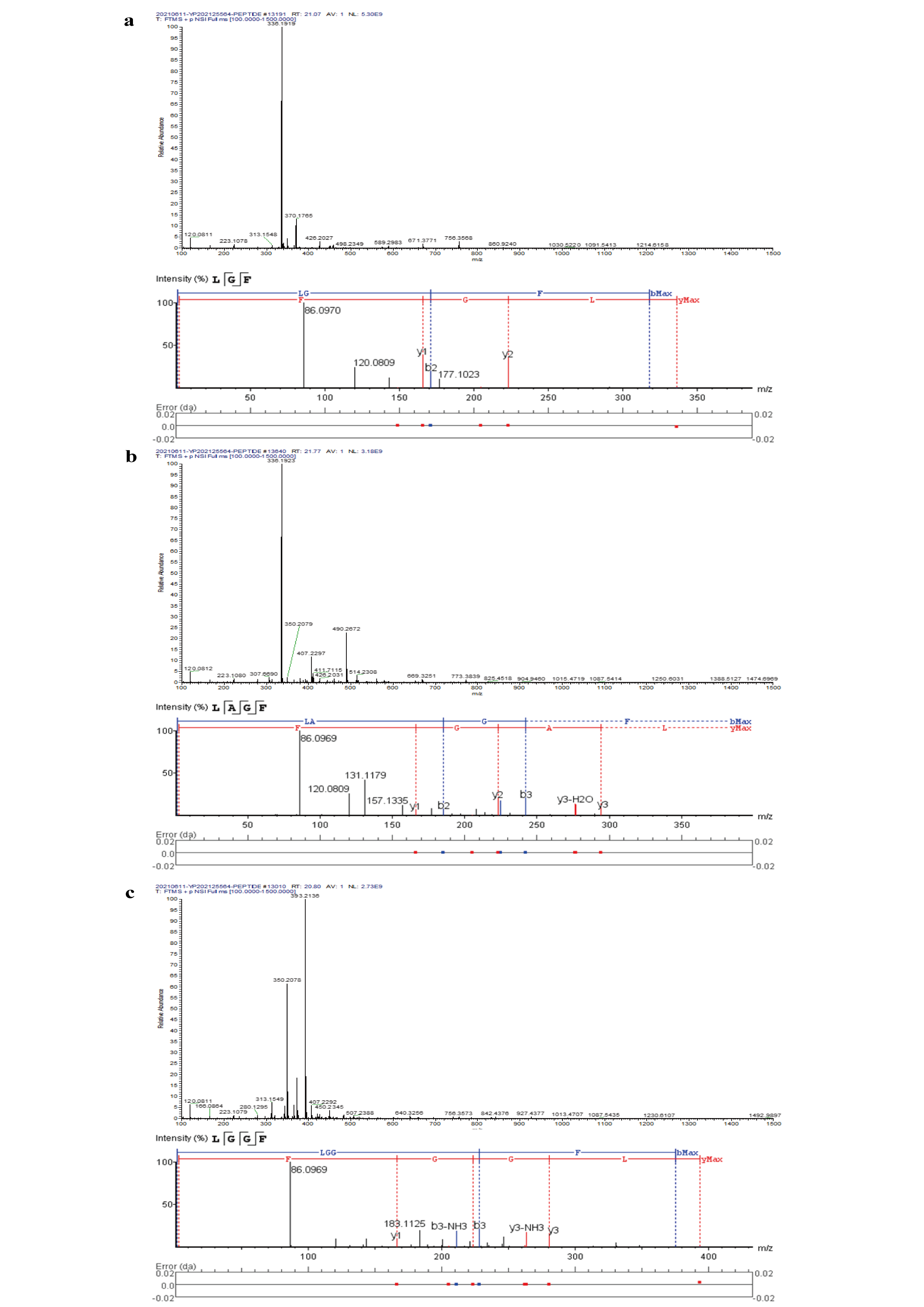

Supplement: Supplementary file 1 [file foods-14-01009-s001.zip › Mass spectrometry sequencing/P1P2P3 Mass Spectrometry Summary.tif]

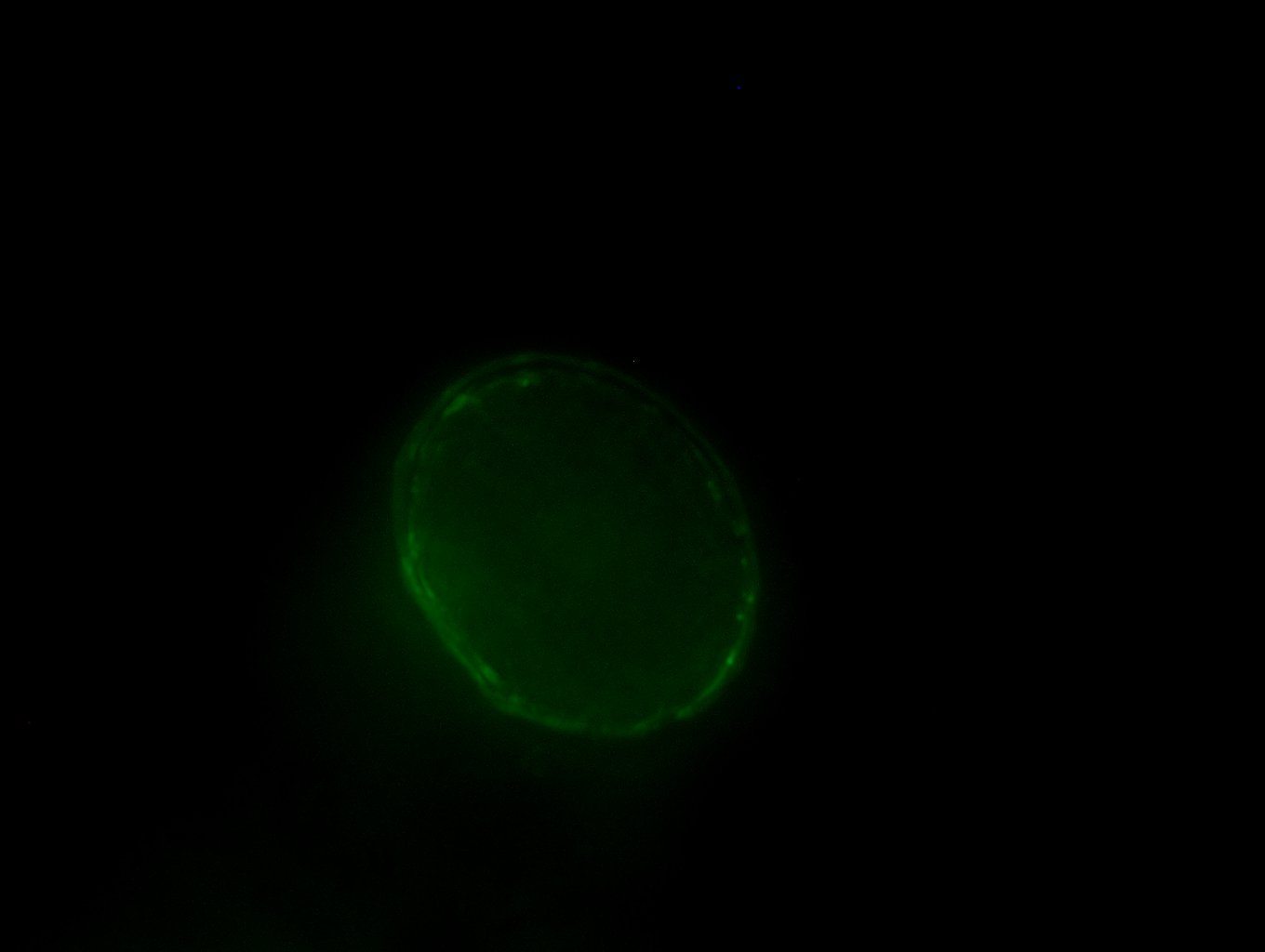

Supplement: Supplementary file 1 [file foods-14-01009-s001.zip › Penetration assessment-Figure S1/Original image of fluorescence microscope/P1/P1 1h-1.jpg]

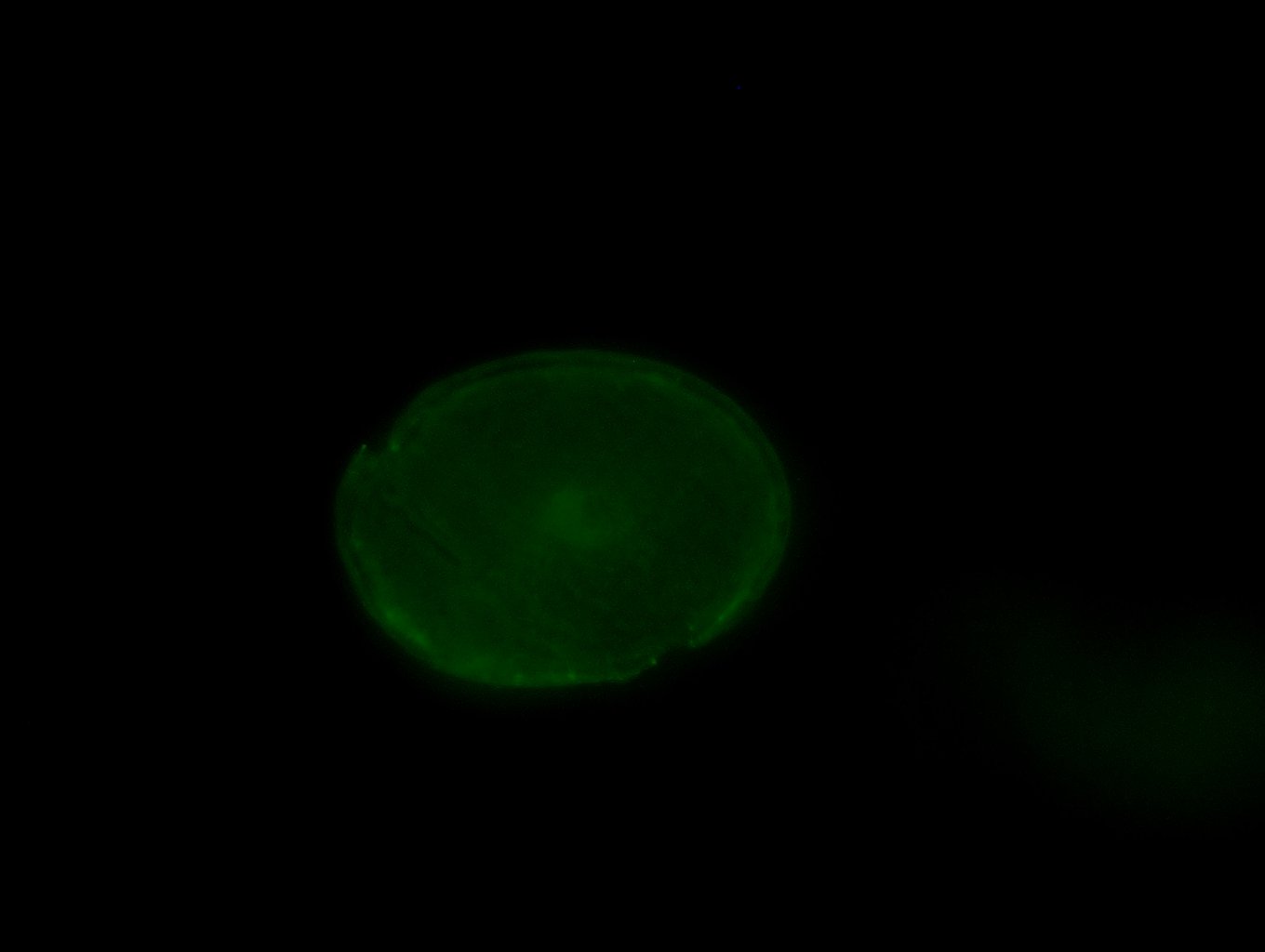

Supplement: Supplementary file 1 [file foods-14-01009-s001.zip › Penetration assessment-Figure S1/Original image of fluorescence microscope/P1/P1 1h-2.jpg]

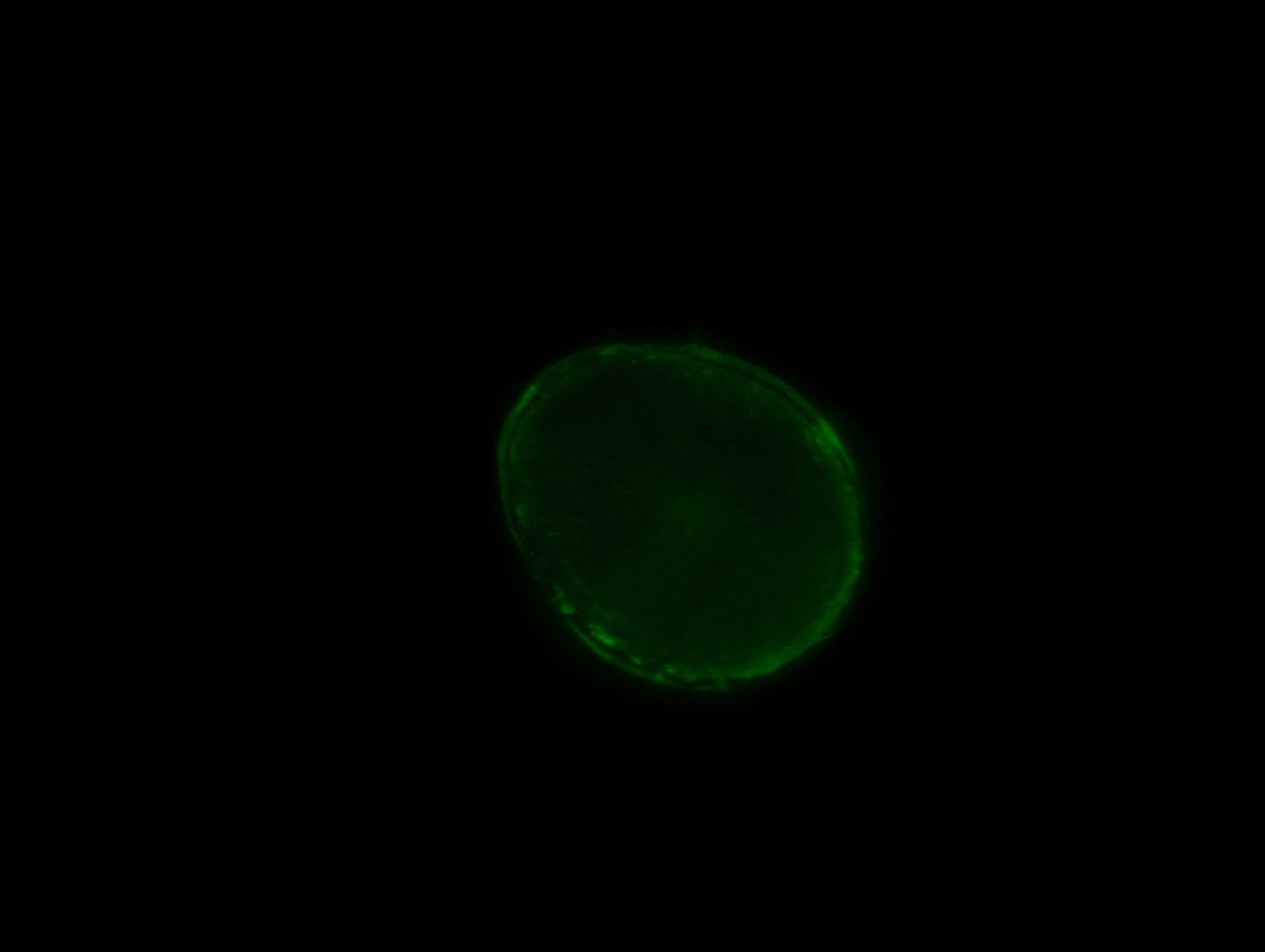

Supplement: Supplementary file 1 [file foods-14-01009-s001.zip › Penetration assessment-Figure S1/Original image of fluorescence microscope/P1/P1 2h-1.jpg]

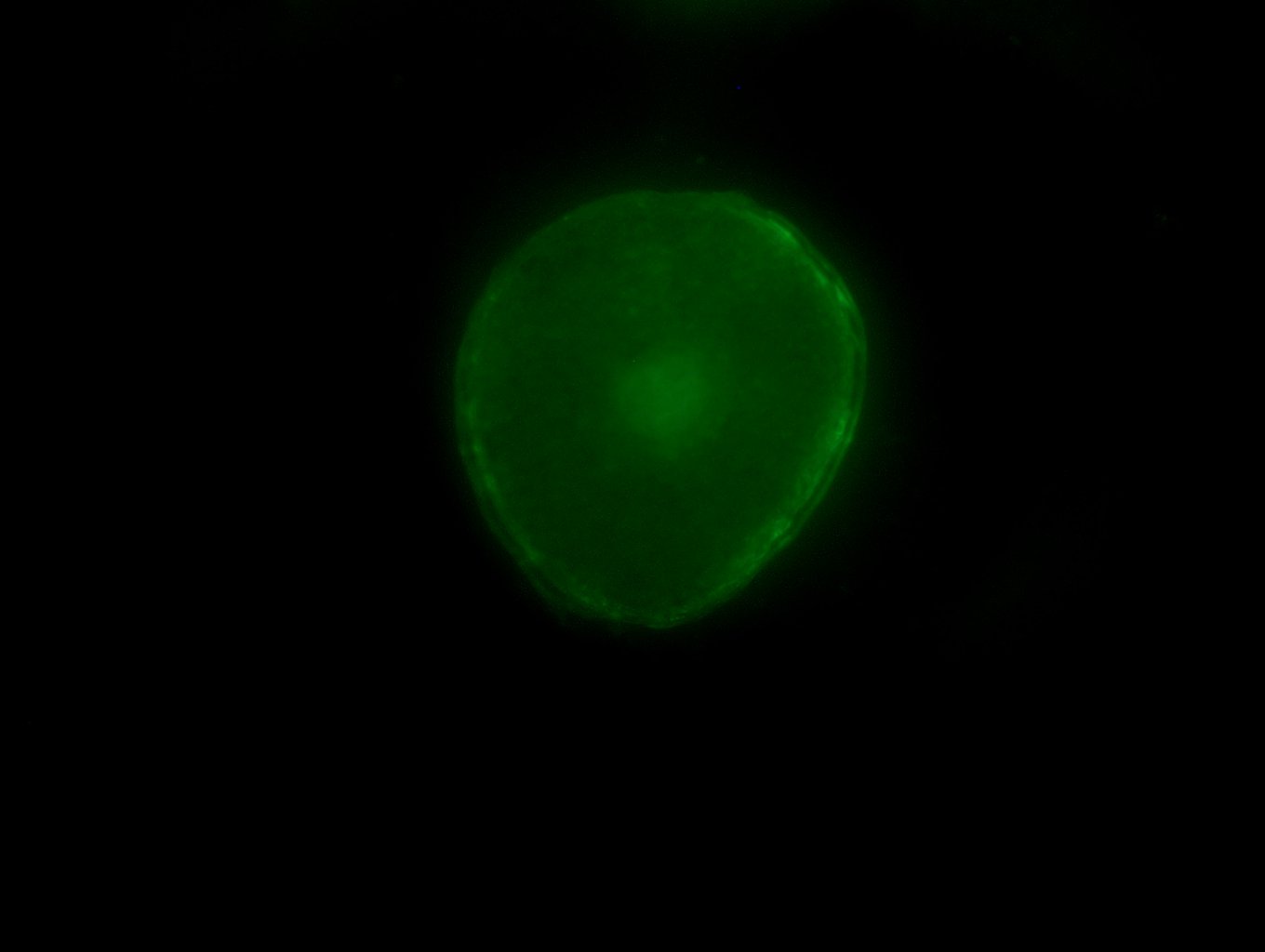

Supplement: Supplementary file 1 [file foods-14-01009-s001.zip › Penetration assessment-Figure S1/Original image of fluorescence microscope/P1/P1 2h-2.jpg]

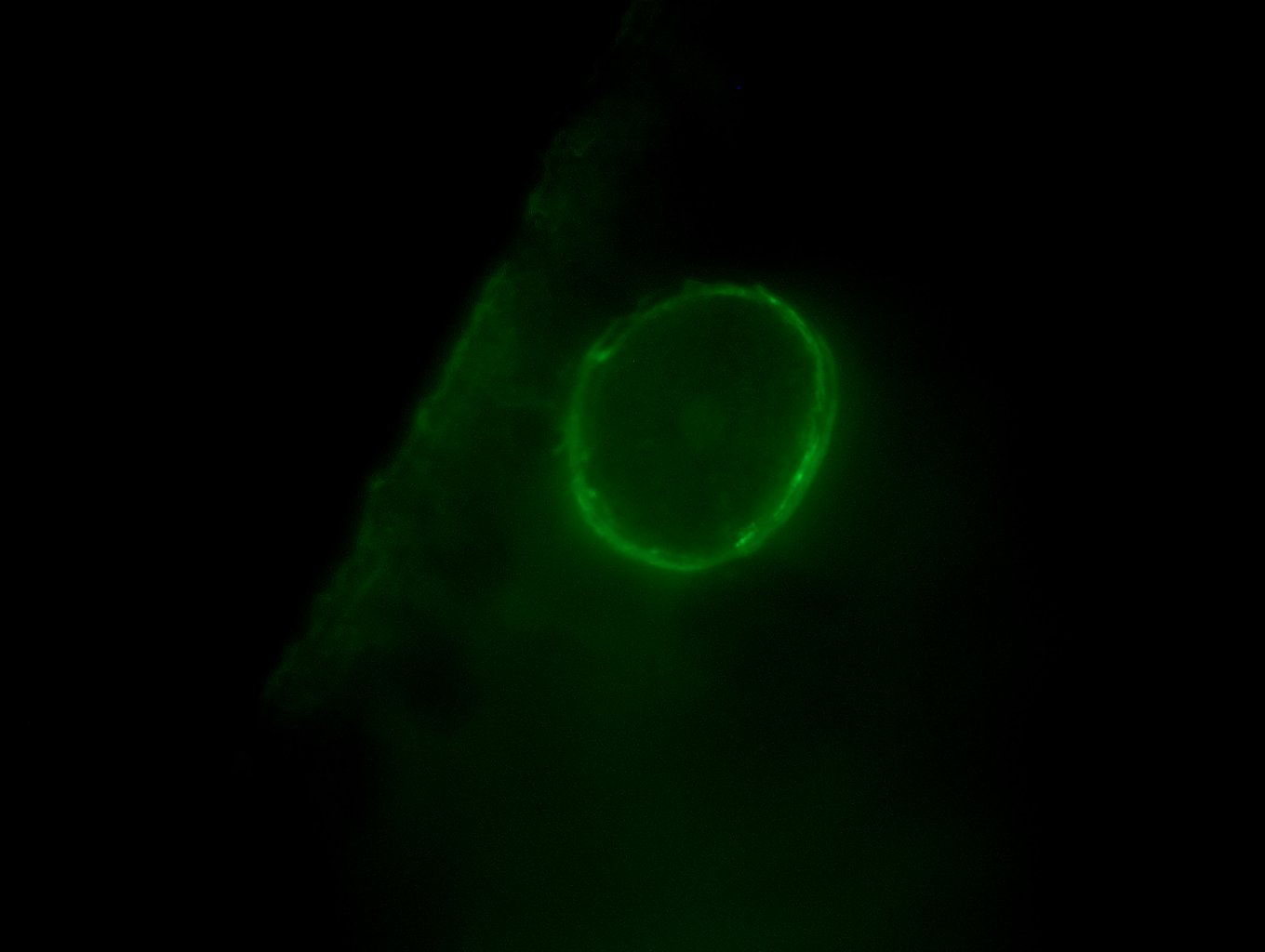

Supplement: Supplementary file 1 [file foods-14-01009-s001.zip › Penetration assessment-Figure S1/Original image of fluorescence microscope/P1/P1 3h-1.jpg]

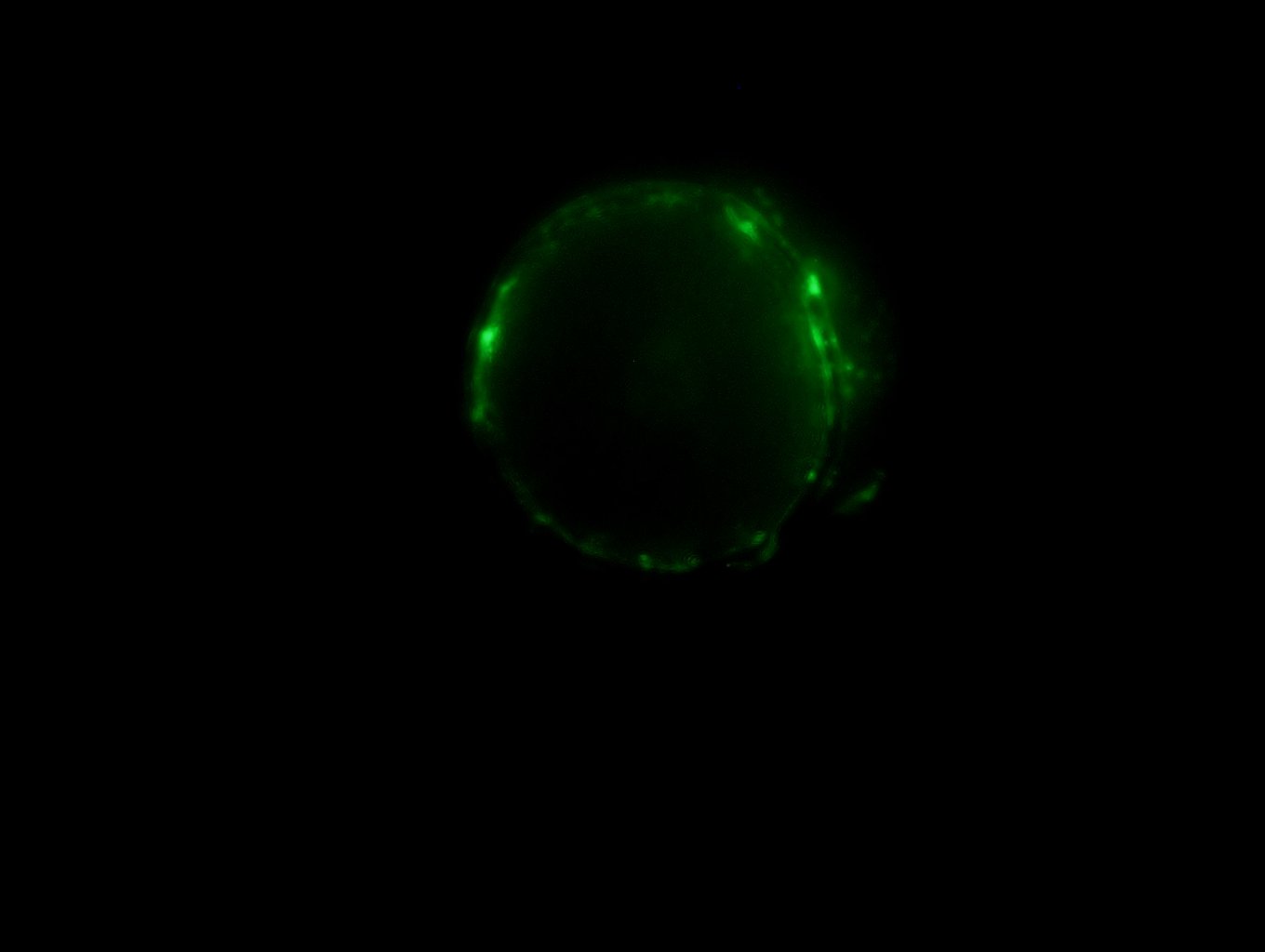

Supplement: Supplementary file 1 [file foods-14-01009-s001.zip › Penetration assessment-Figure S1/Original image of fluorescence microscope/P1/P1 3h-2.jpg]

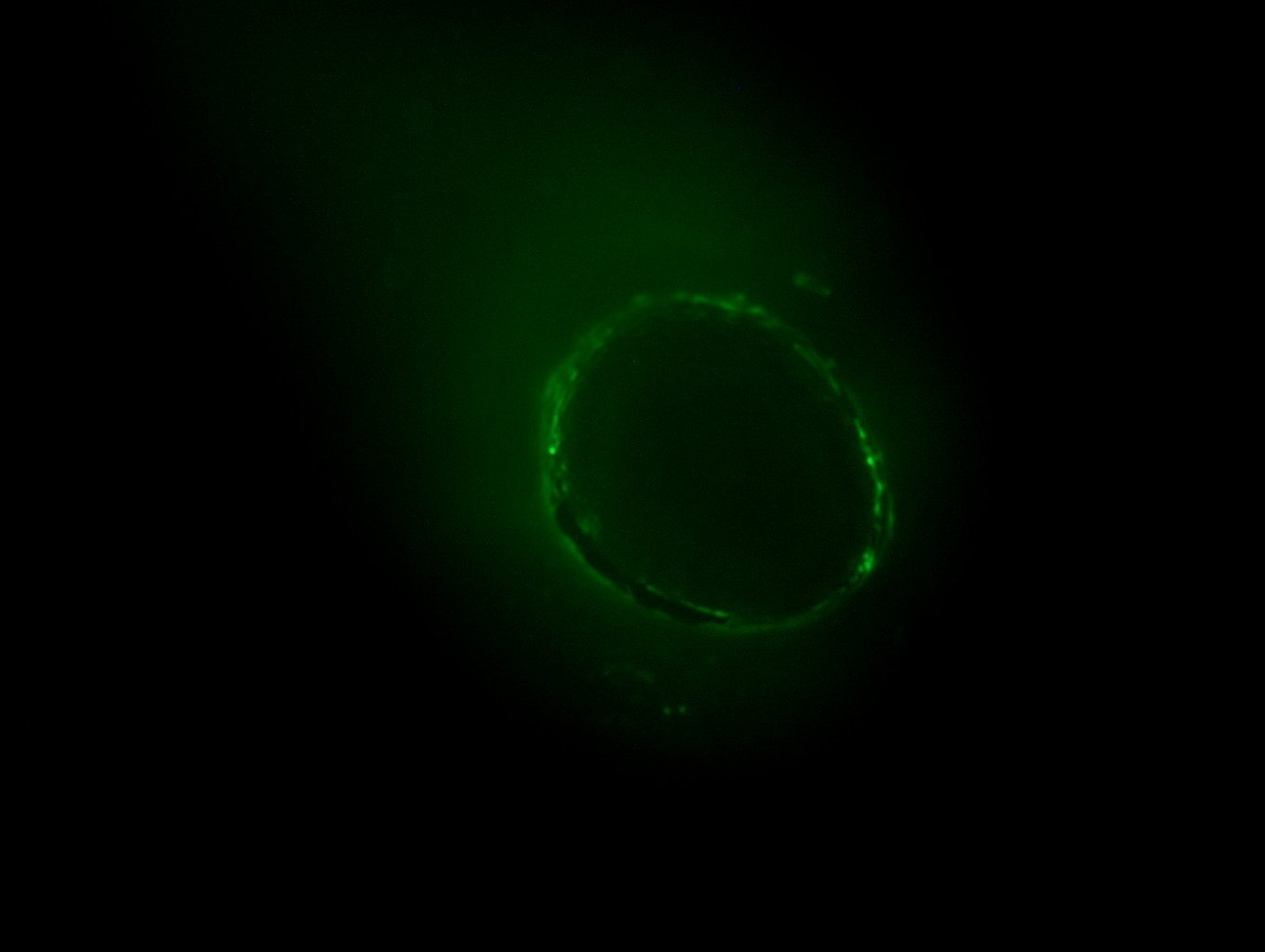

Supplement: Supplementary file 1 [file foods-14-01009-s001.zip › Penetration assessment-Figure S1/Original image of fluorescence microscope/P2/P2 1h-1.jpg]

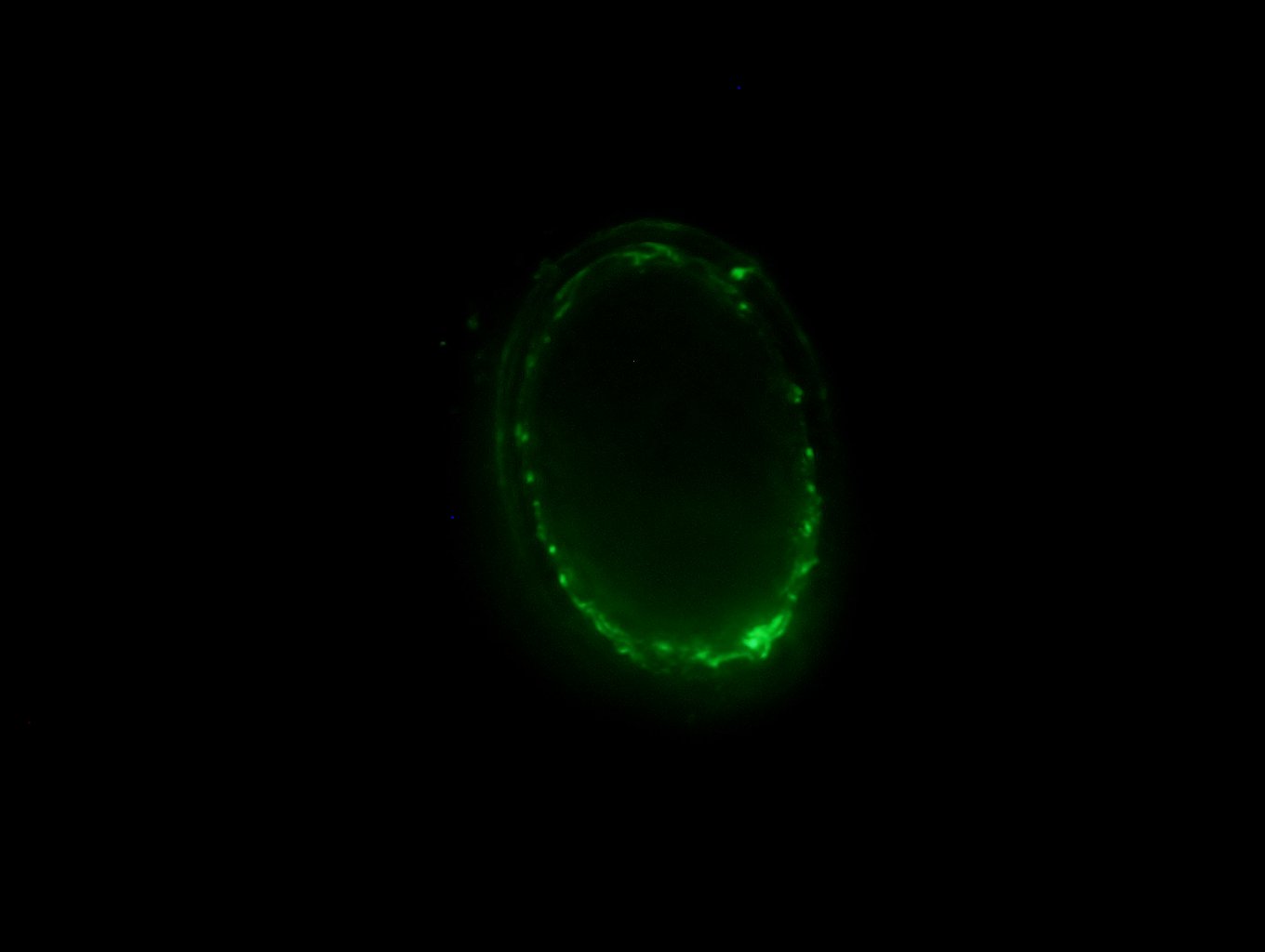

Supplement: Supplementary file 1 [file foods-14-01009-s001.zip › Penetration assessment-Figure S1/Original image of fluorescence microscope/P2/P2 1h-2.jpg]

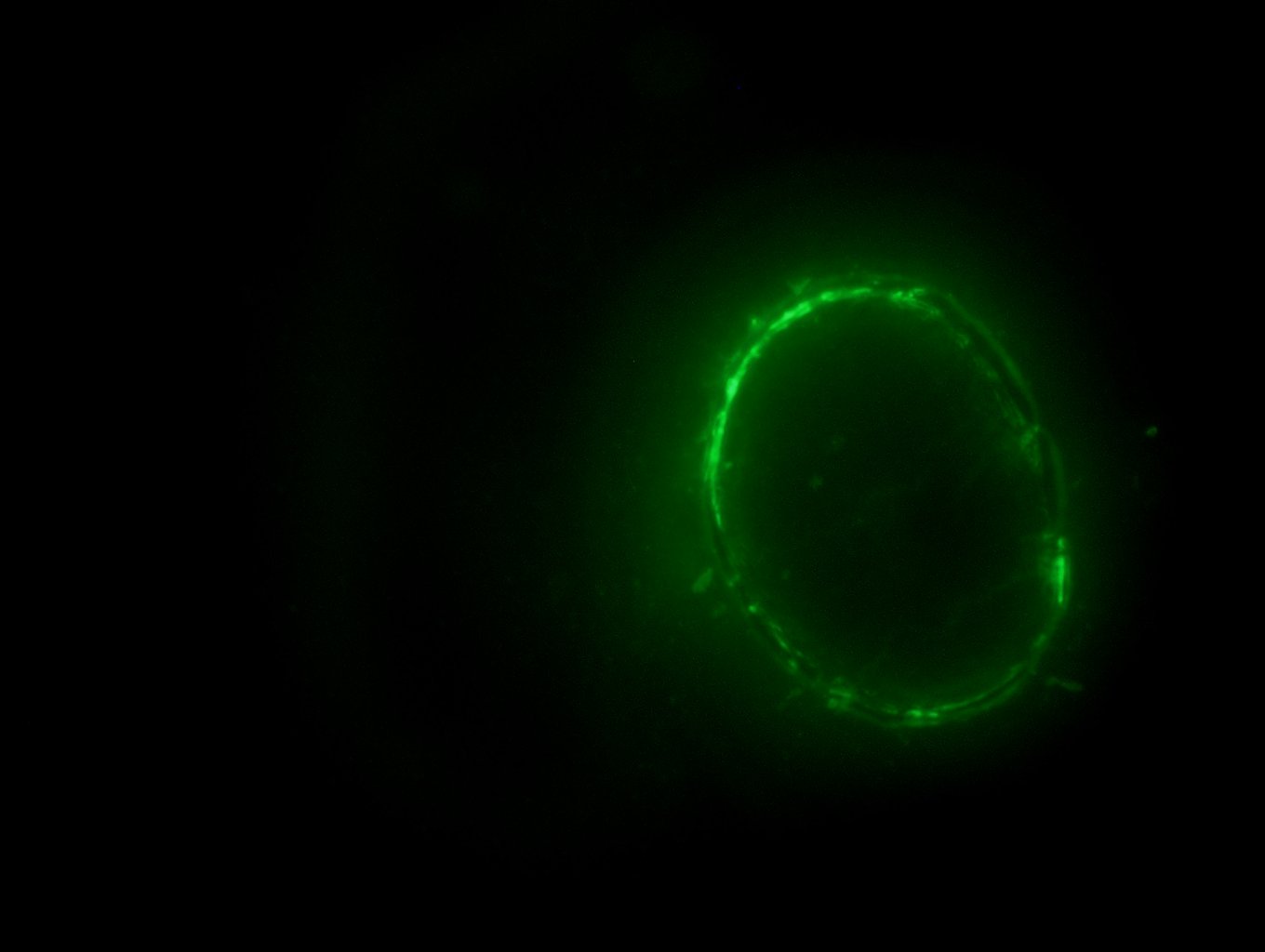

Supplement: Supplementary file 1 [file foods-14-01009-s001.zip › Penetration assessment-Figure S1/Original image of fluorescence microscope/P2/P2 2h-1.jpg]

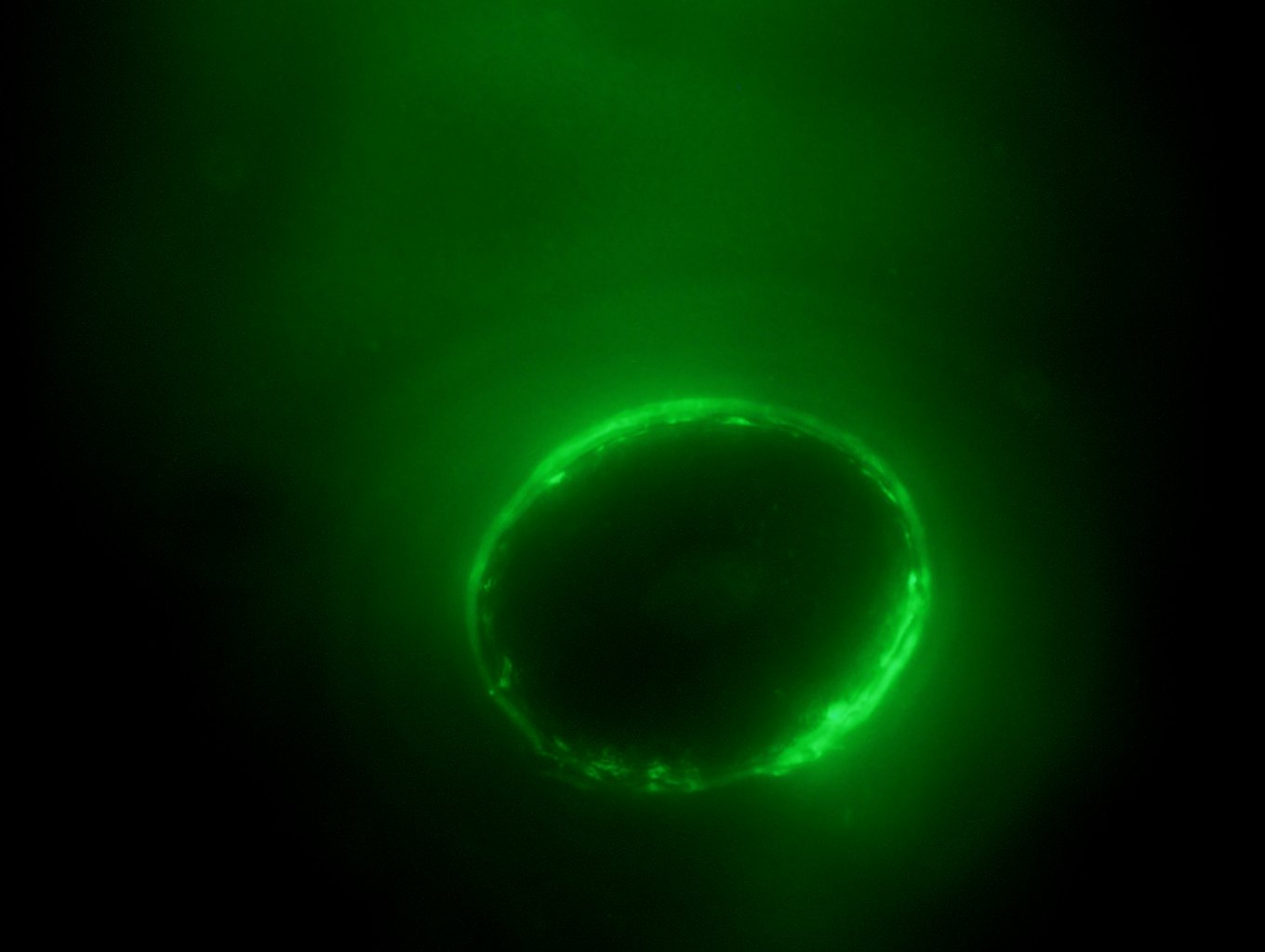

Supplement: Supplementary file 1 [file foods-14-01009-s001.zip › Penetration assessment-Figure S1/Original image of fluorescence microscope/P2/P2 2h-2.jpg]

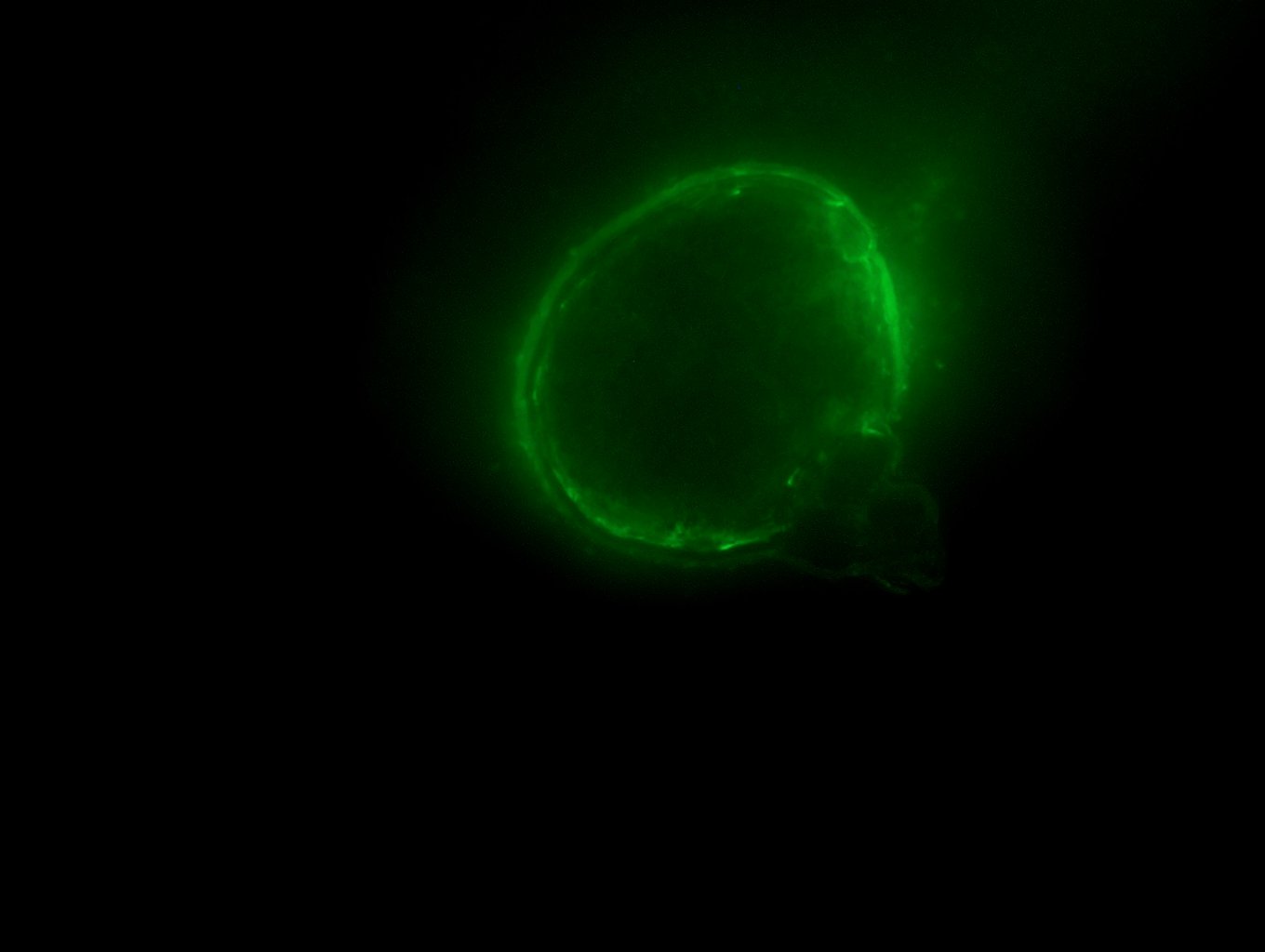

Supplement: Supplementary file 1 [file foods-14-01009-s001.zip › Penetration assessment-Figure S1/Original image of fluorescence microscope/P2/P2 3h-1.jpg]

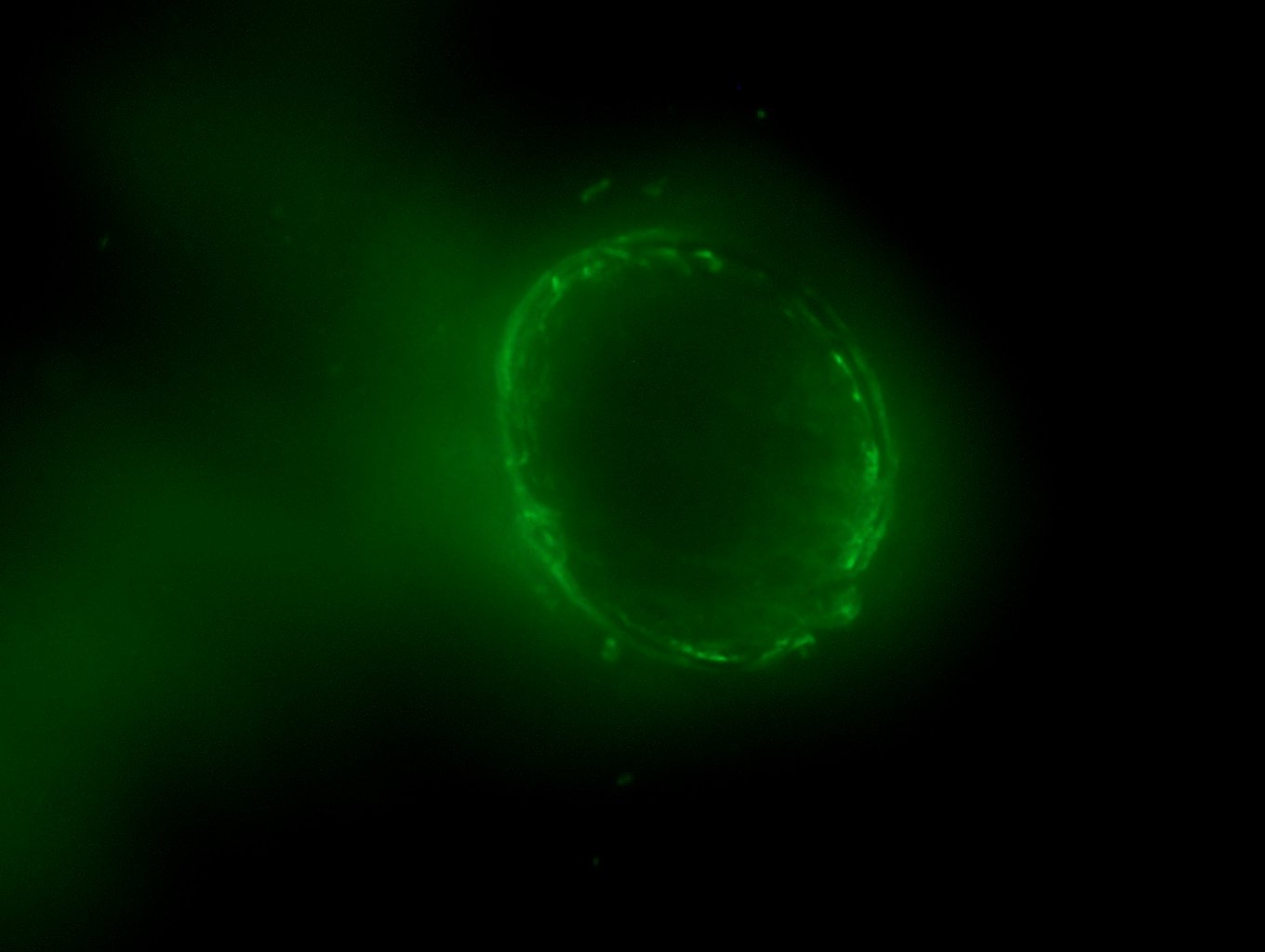

Supplement: Supplementary file 1 [file foods-14-01009-s001.zip › Penetration assessment-Figure S1/Original image of fluorescence microscope/P2/P2 3h-2.jpg]

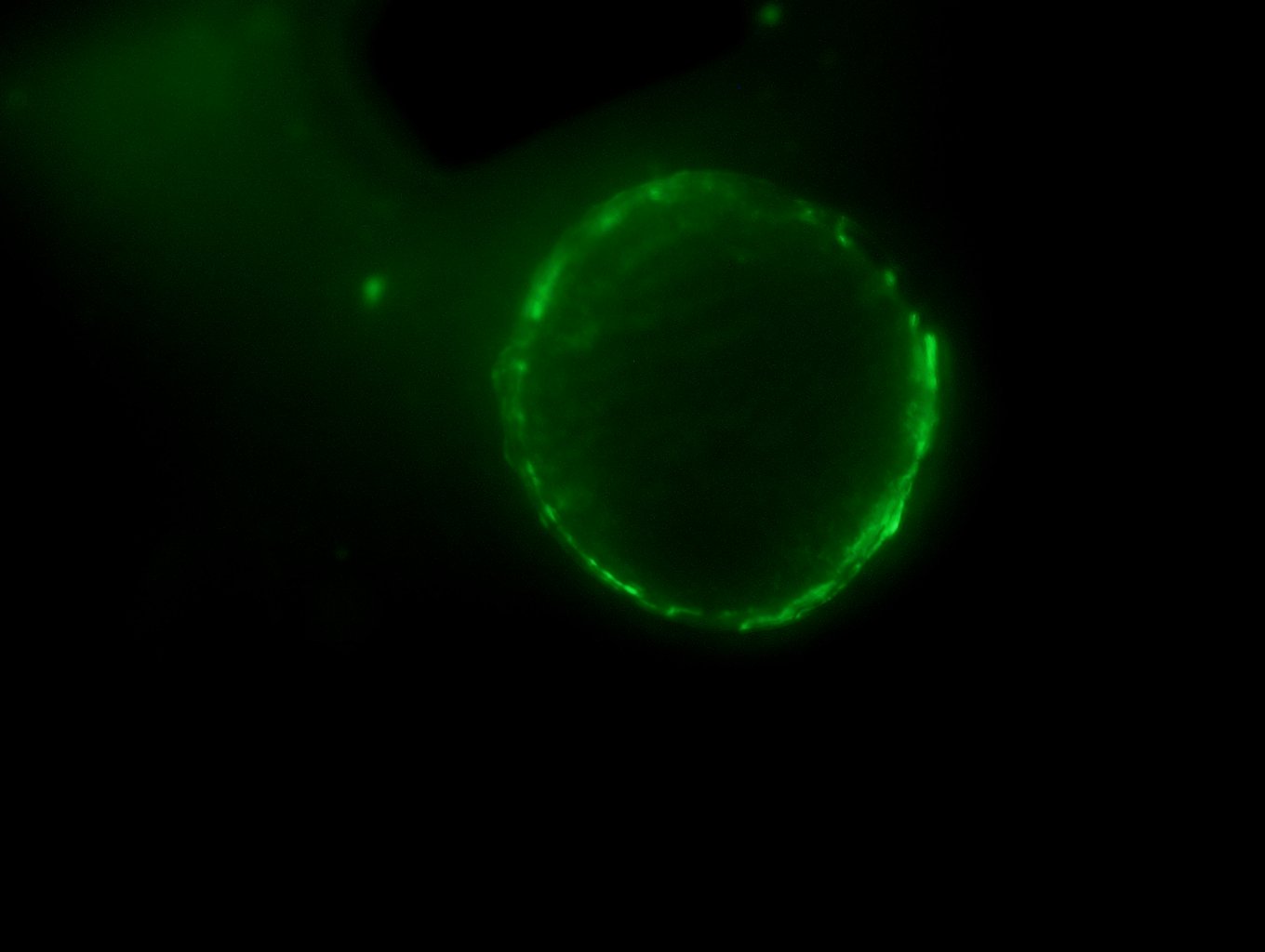

Supplement: Supplementary file 1 [file foods-14-01009-s001.zip › Penetration assessment-Figure S1/Original image of fluorescence microscope/P3/P3 2h-2.jpg]

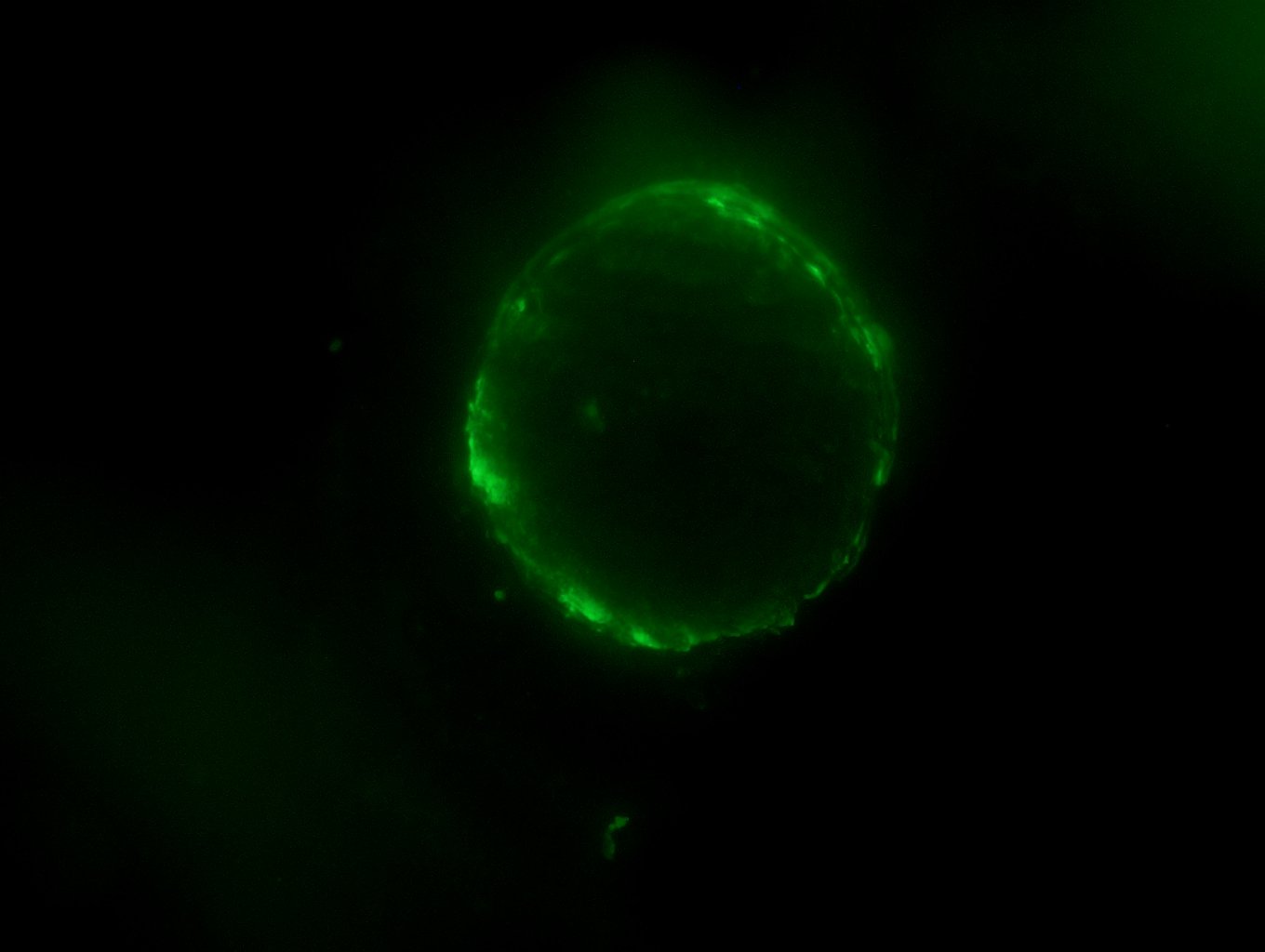

Supplement: Supplementary file 1 [file foods-14-01009-s001.zip › Penetration assessment-Figure S1/Original image of fluorescence microscope/P3/P3 3h-2.jpg]

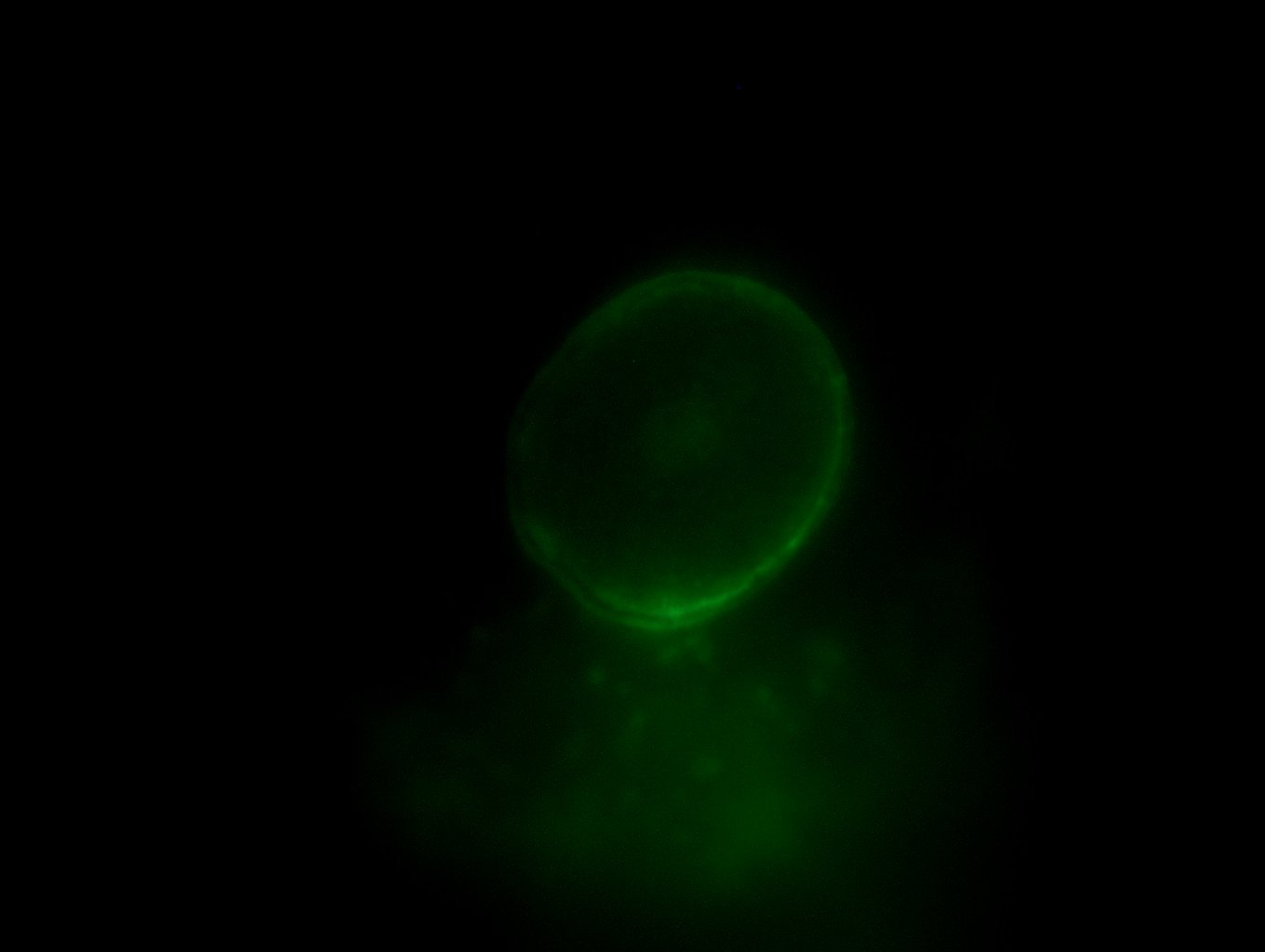

Supplement: Supplementary file 1 [file foods-14-01009-s001.zip › Penetration assessment-Figure S1/Original image of fluorescence microscope/P3/P3 1h-1.jpg]

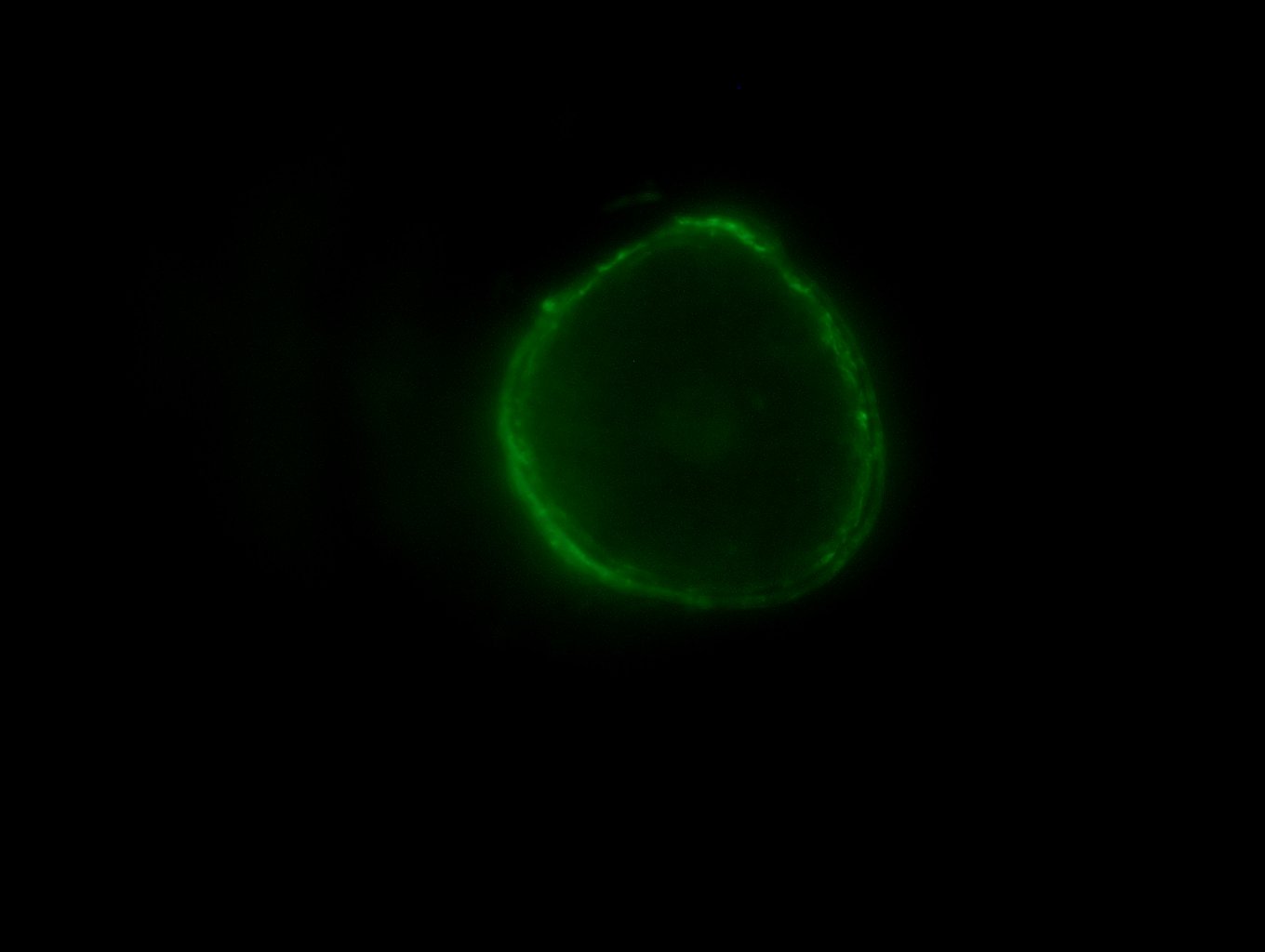

Supplement: Supplementary file 1 [file foods-14-01009-s001.zip › Penetration assessment-Figure S1/Original image of fluorescence microscope/P3/P3 1h-2.jpg]

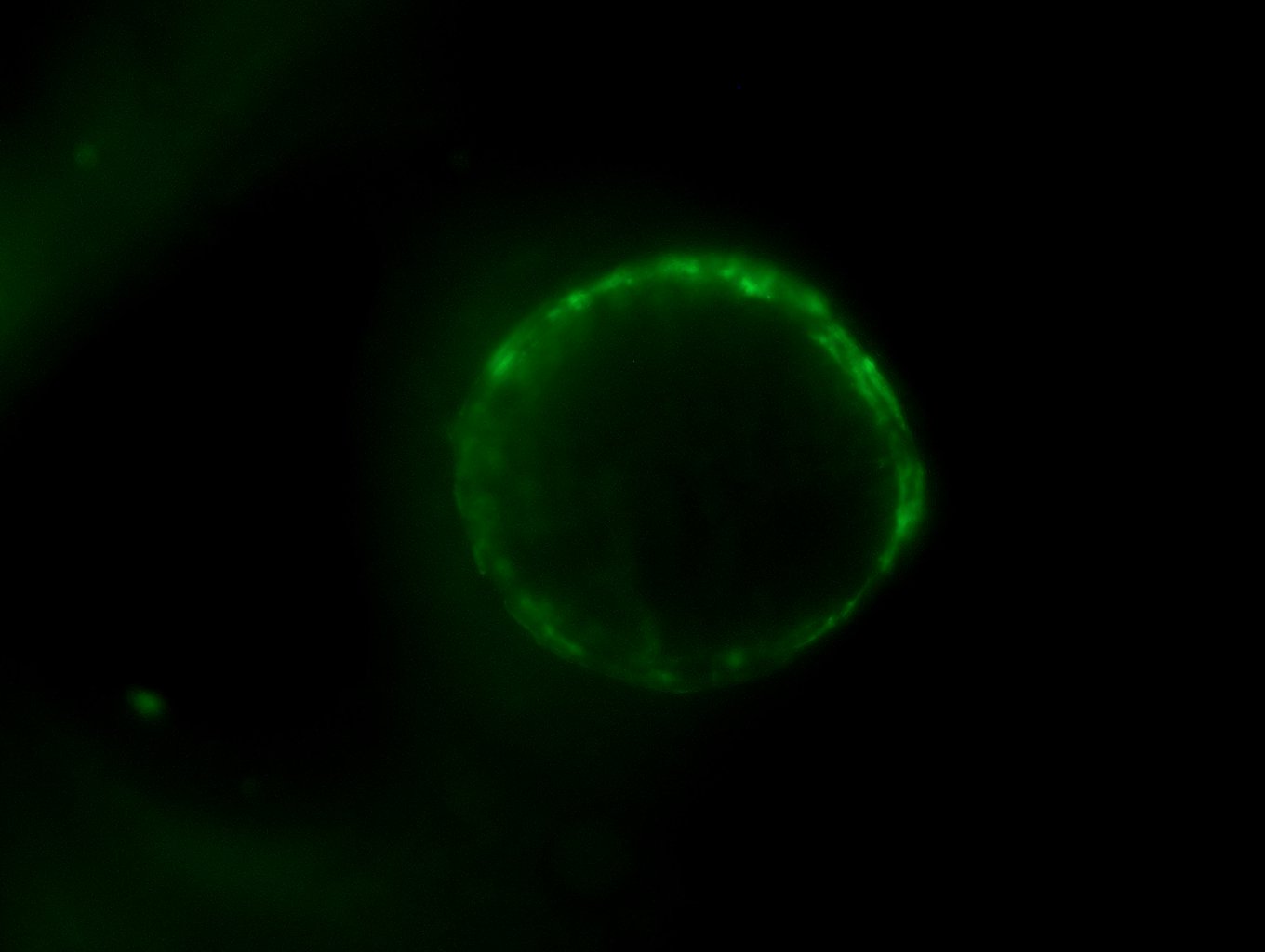

Supplement: Supplementary file 1 [file foods-14-01009-s001.zip › Penetration assessment-Figure S1/Original image of fluorescence microscope/P3/P3 2h-1.jpg]

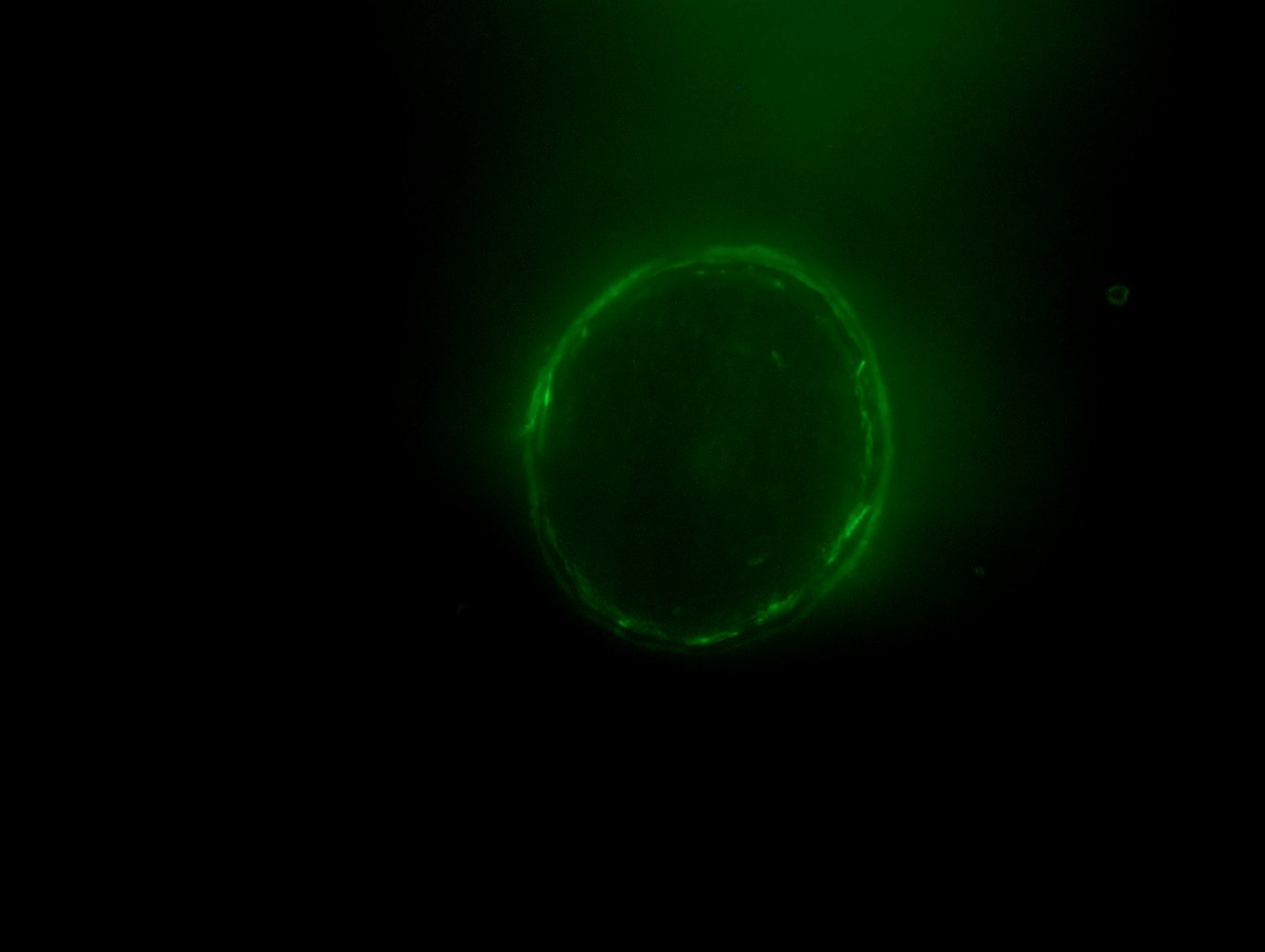

Supplement: Supplementary file 1 [file foods-14-01009-s001.zip › Penetration assessment-Figure S1/Original image of fluorescence microscope/P3/P3 3h-1.jpg]
